# Mechanisms of urodele limb regeneration

**DOI:** 10.1002/reg2.92

**Published:** 2017-12-26

**Authors:** David L. Stocum

**Affiliations:** ^1^ Department of Biology Indiana University−Purdue University Indianapolis 723 W. Michigan St Indianapolis IN 46202 USA

**Keywords:** limb, mechanisms, regeneration, review, urodele

## Abstract

This review explores the historical and current state of our knowledge about urodele limb regeneration. Topics discussed are (1) blastema formation by the proteolytic histolysis of limb tissues to release resident stem cells and mononucleate cells that undergo dedifferentiation, cell cycle entry and accumulation under the apical epidermal cap. (2) The origin, phenotypic memory, and positional memory of blastema cells. (3) The role played by macrophages in the early events of regeneration. (4) The role of neural and AEC factors and interaction between blastema cells in mitosis and distalization. (5) Models of pattern formation based on the results of axial reversal experiments, experiments on the regeneration of half and double half limbs, and experiments using retinoic acid to alter positional identity of blastema cells. (6) Possible mechanisms of distalization during normal and intercalary regeneration. (7) Is pattern formation is a self‐organizing property of the blastema or dictated by chemical signals from adjacent tissues? (8) What is the future for regenerating a human limb?

## 1 INTRODUCTION

Evidence from the fossil record indicates that urodeles (salamanders and newts) of the Permian period (the last period of the Paleozoic era, ∼300 million years ago) were capable of limb regeneration (Fröbisch, Bickelmann, & Witzmann, 2014). How the urodeles evolved the ability to regenerate limbs is a matter of speculation (Brockes, 2015). Although teleost fish can regenerate fins, and larval anurans can regenerate developing limb buds as long as the amputation plane does not pass through differentiated tissue, urodeles are today the only tetrapod vertebrates that can regenerate limbs throughout their life cycle, as well as tails, spinal cord, heart tissue, lens, and retina (Brockes & Kumar, 2008; Nacu & Tanaka, 2011; Stocum & Cameron, 2011; for reviews). Although adult mice and humans can regenerate the distal tip of the terminal phalanges, their limbs do not regenerate after amputation at more proximal levels. In humans, the remedies for such amputations are replants, allotransplants, or bionic appendages.

Some gene activities, progenitor cells, and tissue interactions in regenerating salamander limbs are similar to those of regenerating mouse digit tips (for reviews see Simkin et al., 2015; Zielens, Ransom, Leavitt, & Longaker, 2016). These similarities have encouraged the idea that mammals have retained a latent ancestral genetic circuitry for appendage regeneration that might be activated by appropriate interventions and applied to the goal of regenerating a human limb. Research on the mechanisms of urodele limb regeneration is central to this goal, and continues to expand within the broader context of regenerative biology and medicine. This paper is intended as a broad review of what we know—and do not know—about the basic biology of urodele limb regeneration.

## 2 PHASES AND STAGES OF LIMB REGENERATION

Spallazani (1768) was the first to provide a description of limb regeneration, in adult newts (Dinsmore, 1991). Systematic studies on limb development and regeneration, however, did not begin until late in the 19th century. In 1901, T. H. Morgan reviewed our conceptual and experimental knowledge of regeneration in his classic book *Regeneration*. Amphibian limb regeneration studies were numerous worldwide during the first half of the 20th century and continued to expand as a part of experimental developmental biology. These studies began with the anatomy, morphology, and histology of regeneration, followed by experimental manipulations to reveal interactions among tissues during regeneration, and have continued into the 21st century focused on the molecular biology and immunology of regenerative mechanisms. A substantial number of texts on limb regeneration have summarized the information that has come out of these later studies (Carlson, 2007; Goss, 1969; Mattson, 1976; Needham, 1952; Polezhaev, 1972; Schmidt, 1968; Stocum, 1995, 2012; Tsonis, 1996; Vorontsova & Liosner, 1960; Wallace, 1981).

**Figure 1:**
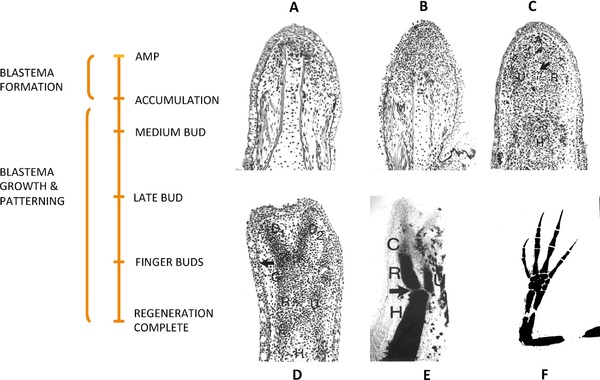
Phases, stages, and longitudinal sections of forelimb regeneration in a urodele larva (*Ambystoma maculatum*) after amputation through the mid‐stylopodium of the forelimb. Longitudinal sections at various stages of regeneration: (A) accumulation blastema, or early bud; (B) medium bud; (C) late bud, with arrow pointing to a blood vessel, and prominent AER; (D) notch, indicating anlagen of anterior two digits (D1, D2), distal humerus, and radius (R) and ulna (U). The arrow indicates the re‐forming basement membrane. (E) Two‐fingerbud whole mount stained with methylene blue. H, humerus; R, radius; U, ulna; C, carpal region. The arrow points to the elbow joint. (F) Methylene blue stained whole mount of fully regenerated limb. After Stocum (2012, Chapter 8)

Thornton (1968) reviewed the histological and morphological events of limb regeneration in detail. These events can be arbitrarily divided into two overlapping phases (Fig. 1). The first phase is the breakdown of stump tissues (histolysis) at the amputation site to yield a collection of undifferentiated progenitor cells called the accumulation or early bud blastema similar in structure to the early embryonic limb bud. The formation of a limb bud‐like blastema in continuity with more proximal differentiated tissues is an injury response unique to urodeles. The accumulation blastema is avascular and lacks innervation. The second phase is the development of the accumulation blastema by coordinated growth, morphogenesis, and differentiation to replace the amputated structures. Initiation of this phase coincides with the re‐vascularization and re‐innervation of the accumulation blastema.

The developmental phase of limb bud regeneration can be subdivided into several morphological stages, each characterized by its own unique histological structure and pattern of gene expression: a conical medium bud, a larger late bud or palette during which redifferentiation of the amputated segments is initiated in a proximal to distal and anterior to posterior order, culminating in the appearance of the digits. Amputated limbs follow the “rule of distal transformation”; i.e., they regenerate only those parts distal to the level of amputation, even when the proximodistal (PD) polarity of the limb is reversed by implanting its distal end in a pocket made in the flank and amputating through the stylopodium. The blastemas that form on each cut end of the stylopodium both regenerate all the parts normally distal to the level of the cut (Butler, 1955; Deck & Riley, 1958; Dent, 1954). Urodele limbs can regenerate after repeated amputations, and all four limbs will regenerate if amputated simultaneously. Adult newt limbs regenerate with high morphological fidelity after one amputation, but repeated amputations lead to progressively greater numbers of abnormalities in the regenerates (Dearlove & Dresden, 1976). Whether this is a general rule in other urodele larvae or adults is not known.

## 3 FORMATION OF THE ACCUMULATION BLASTEMA

The accumulation blastema forms as the result of three processes: (1) formation of a wound epidermis to close the wound by epidermal migration from the cut edges of the skin; (2) generation of progenitor (“blastema”) cells by histolysis and the release of dedifferentiated and/or resident stem/progenitor cells; and (3) blastema cell migration and aggregation under an apical thickening of the wound epidermis, the apical epidermal cap (AEC). The AEC is a distal signaling center for promoting blastema cell mitosis that is analogous to the apical ectodermal ridge (AER) of amniote limb buds.

### 3.1 The wound epidermis

Immediately after amputation the wound is sealed by a thrombin‐catalyzed blood clot. The epidermal basal cells at the edge of the cut skin lose their intercellular junctions and hemidesmosomal junctions that adhere them to the basement membrane and migrate through the clot to close the wound within a few hours. The migrating cells do not divide (Hay & Fischman, 1961), but a zone of dividing epidermal cells proximal to the wound edge supplies a continual stream of migrating cells (Lash, 1955; Repesh & Oberpriller, 1978, 1980). Fibronectin in the clot is the adhesive substrate for the migrating epithelial cells (Donaldson & Mason, 1977; Donaldson, Mahan, Yang, & Crossin, 1991; Repesh, Furcht & Smith, 1981). As the accumulation blastema forms, the wound epidermis thickens at its apex to form the AEC.

The wound epidermis expresses two antigens designated WE3 and WE6 that are thought to be actin‐binding proteins and to regulate secretion and/or ionic composition (Castilla & Tassava, 1992; Estrada, Park, Castilla, & Tassava, 1993; Goldhamer, Tomlinson, & Tassava, 1989; Tassava & Acton, 1989; Tassava, Castilla, Arsanto, & Thouveny, 1993; Tassava, Johnson‐Wint, & Gross, 1986). These antigens are not expressed in uninjured epidermis, indicating that they are specific to regeneration. The gene for *Sp9*, a transcription factor that plays a key role in amniote limb development by its positive regulation of fibroblast growth factor 8 (Fgf8) expression (Kawakami et al., 2004), is also expressed in the wound epidermis of regenerating axolotl limbs and may be involved in formation of the AEC (Satoh, Cummings, Bryant, & Gardiner, 2010a). Epidermal ion channels generate early signals obligatory for blastema formation, including Na^+^ influx/H^+^ efflux (Adams, Masi, & Levin, 2007; Jenkins, Duerstock, & Borgens, 1996). How these early signals are linked to secretory functions of the wound epidermis and the subsequent events of histolysis and dedifferentiation is not clear, but may involve upregulation of nitric oxide signals in the epidermis and stimulation of a rise in cytosolic Ca^2+^ that results in the localization of protein kinase C to the plasma membrane, where it is activated by diacylglycerol to regulate transcription (Rao et al., 2009). Many other genes are upregulated in the wound epidemis, the functions of which have not yet been determined (Campbell et al., 2011).

### 3.2 Histolysis

Histolysis is the degradation of extracellular matrix (ECM) of limb tissues local to the amputation surface by proteolytic enzymes, particularly lysosomal acid hydrolases and matrix metalloproteinases (MMPs) (Dresden & Gross, 1970; Ju & Kim, 1998; Miyazaki, Uchiyawa, Imokawa, & Yoshizato, 1996; Park & Kim, 1999; Santosh et al., 2011; Schmidt, 1968; Yang & Bryant, 1994; Yang, Gardiner, & Bryant, 1999). Histolysis liberates fibroblasts from the dermis, interstitial connective tissue of muscle, periosteum, and nerve sheath, as well as Schwann cells from the peripheral nerves. Myofibers fragment at their cut ends and break up into mononucleate cells while simultaneously releasing Pax7^+^ muscle stem cells, the satellite cells (Hay, 1959; Sandoval‐Guzman et al., 2014; Thornton, 1938a, b). MMPs also prevent reassembly of a basement membrane, thereby ensuring contact between the wound epidermis and the underlying tissues. The importance of MMPs to histolysis is underscored by the failure of blastema formation in amputated newt limbs treated with the MMP inhibitor GM6001 (Vinarsky, Atkinson, Stevenson, Keating, & Odelberg, 2005). The wound epidermis and the AEC are major sources of MMPs (Godwin, Pinto, & Rosenthal, 2013) and also function to eliminate cellular and particulate debris generated by tissue destruction and the bactericidal activity of neutrophils and macrophages (Singer & Inoue, 1964; Singer & Salpeter, 1961).

Histolysis of stump tissue continues until the medium bud stage, when it declines due to the activity of tissue inhibitors of metalloproteinases (TIMPS) (Santosh et al., 2011; Stevenson, Vinarsky, Atkinson, Keating, & Odelberg, 2006). TIMP1 is upregulated when MMP levels approach maximum, and exhibits a spatial pattern of expression congruent with patterns of MMP expression in the wound epidermis, proximal epidermis, and internal tissues undergoing disorganization. How MMP and TIMP expression patterns are coordinated is unknown.

### 3.3 Dedifferentiation

#### 3.3.1 Transcriptional changes

The cells liberated by histolysis are mononucleate progenitor (blastema) cells that are a mixture of resident stem/progenitor cells and dedifferentiated cells. Blastema cells resemble the mesenchymal cells of the limb bud, with large nuclei, sparse cytoplasm, large numbers of free ribosomes, and a vesiculated endoplasmic reticulum (Hay, 1958, 1959; Lentz, 1967). They exhibit intense DNA, RNA, and protein synthesis (Anton, 1965; Bodemer, 1962; Bodemer & Everett, 1959; Hay & Fischman, 1961; Morzlock & Stocum, 1971). Blastema cells appear within 2–3 days post‐amputation in larval urodeles and within 4–5 days in adult newts. As they accumulate, new capillaries and nerve axons regenerate from their cut ends into the accumulation and the wound epidermis thickens into the AEC.

Dedifferentiation involves epigenetic nuclear reprogramming that suppresses the transcription of differentiation genes, while activating transcription of genes and translation of proteins associated with stemness, reduction of cell stress, and remodeling internal structure (Gardiner & Bryant, 2002; Geraudie & Ferretti, 1998; Rao et al., 2009). Inhibition of these transcriptional changes by actinomycin D does not affect histolysis, but does prevent or retard dedifferentiation, leading to regenerative failure or delay (Carlson, 1969). Dedifferentiated cells express a more limb bud‐like ECM in which type II collagen synthesis is suppressed, type I collagen synthesis remains the same, and fibronectin, tenascin, and hyaluronate accumulate (Ashahina, Obara, & Yoshizato, 1999; Gulati, Zakewski, & Reddi, 1983; Mescher & Munaim, 1986; Onda, Poulin, Tassava, & Chiu, 1991). A temporary “transitional matrix” has been described during early blastema formation in amputated newt limbs that may facilitate the cellularization of myofibers and sustain dedifferentiation of the resulting mononucleate cells (Calve, Odelberg, & Simon, 2010).

The molecular details of transcriptional regulation during dedifferentiation are only partly known. Approaches to examining gene activity during regeneration involve quantifying the expression of individual genes and global analyses of gene and protein expression. Individual genes associated with progenitor status that are upregulated during blastema formation are *msx1*, *msx2*, *nrad*, *rfrng*, and *notch* (Cadinouche, Liversage, Muller, & Tsifildis, 1999; Carlson, Bryant, & Gardiner, 1998; Crews et al., 1995; Géraudie & Ferretti, 1998, for a review; Koshiba, Kuroiwa, Yamamoto, Tamura, & Ide, 1998; Shimizu‐Nishikawa, Tsuji, & Yoshizato, 2001; Simon et al., 1995). *Msx1* inhibits myogenesis (Woloshin et al., 1995) and its forced expression in mouse C2C12 myotubes causes cellularization and reduced expression of muscle regulatory proteins (Odelberg, Kollhof, & Keating, 2001). Inhibiting *msx1* expression with anti‐*msx* morpholinos in cultured newt myofibers prevents their cellularization and reduces their expression of muscle regulatory proteins (Kumar, Velloso, Imokawa, & Brockes, 2004). *Nrad* expression is correlated with muscle dedifferentiation (Shimizu‐Nishikawa et al., 2001), and *Notch* is a major mediator of stem cell self‐renewal (Lundkvist & Lendahl, 2001).

A number of differentially upregulated genes in the early axolotl limb blastema were identified by subtractive hybridization (Gorsic, Majdic, & Kornel, 2008). Most of these genes fell into the categories of metabolism, cell physiological process, cell cycle regulation, and protein synthesis and transport. Subtractive hybridization was also used to compare transcript expression after amputation at a regeneration‐competent versus a regeneration‐deficient stage of *Xenopus* limb bud development (King et al., 2003). This study identified three categories of cDNA clones: clones expressed at both competent and deficient blastemas, clones with highest expression in regeneration‐competent blastemas, and clones with highest expression in regeneration‐deficient blastemas.

Microarray and RNA‐Seq analysis of regenerating axolotl limbs has identified suites of genes encoding progenitor cell markers, stage‐specific genes, and genes regulated by neural signals (Knapp et al., 2013; Looso et al., 2013; Mercer et al., 2012; Monaghan et al., 2009, 2012; Stewart et al., 2013; Vascotto, Beug, Liversage, & Tsilfildis, 2005; Voss et al., 2015). Bryant, et al. (2017) have assembled an axolotl transcriptome that identifies transcripts enriched in individual limb tissues and which distinguishes blastemas from differentiated limb tissues. This study revealed two highly upregulated genes, the RNA binding protein gene *cirbp* and the serine protease inhibitor gene *kazald1*. Cirbp has a cytoprotective role in limb regeneration, whereas knockdown or overexpression of the kazald1 protein impairs regeneration.

Since not all transcripts are translated into proteins, proteomic studies are also important to the analysis of regenerative mechanisms. Franco et al. (2013) have reviewed proteomic studies of regeneration in a wide variety of organisms that have high regenerative ability. Changes in the proteome during blastema formation in regenerating axolotl, newt, and developing and adult *Xenopus* limbs have been investigated by Rao et al. (2009, 2014), Looso et al. (2013), and King, Mescher, and Neff (2009). These studies have revealed patterns of upregulation and downregulation of proteins in various biological process categories such as signaling, transcription, translation, cytoskeleton, ECM, metabolism and cell cycle. The highly upregulated and downregulated genes and proteins identified in genomic, transcriptomic, and proteomic studies can now be the focus for specific analysis of regenerative pathways (Jhamb et al., 2011).

Three of the six transcription factor genes (*klf4*, *sox2*, *c‐myc*) used to reprogram mammalian adult somatic cells to induced pluripotent stem cells (iPSCs) (Takahashi et al., 2007; Yu, Vodyanik et al., 2007) were found to be upregulated during blastema formation in regenerating newt limbs, and also during lens regeneration (Maki et al., 2009). The Lin 28 protein, the product of a fourth transcription factor gene used to derive iPSCs (Yu, Vodyanik et al., 2007), is also upregulated during blastema formation in regenerating axolotl limbs (Rao et al., 2009). Blastema cells, however, are not pluripotent. In a comparison of iPSCs and regenerating *Xenopus* limb and tail buds, Christen, Robles, Raya, Paramonov, and Izpisua Belmonte (2010) found that some pluripotency genes—*Oct4*, *Sox2*, *c‐Myc*, *klf4*, *tert*, *Sall4* and others—were expressed before and during regeneration, but were not upregulated to the extent expected for pluripotency. Thus, although these factors may play a role in nuclear reprogramming during limb regeneration, they may not be expressed to the degree required to achieve pluripotency, or other factors must exist (or be lacking) that prevent reprogramming to this extreme.

Micro RNAs (miRNAs), small non‐coding RNAs that downregulate gene expression by binding to complementary sequences in the 3′ untranslated region of target mRNAs, are expressed in a gene regulatory circuit in regenerating axolotl limbs and fish fins (King & Yin, 2016). A specific miRNA identified in the axolotl regeneration blastema is miR‐21, which targets the gene *Jagged1*, and may downregulate this gene to facilitate transition from a proliferative state to cell fate commitment (Holman, Campbell, Hines, & Crews, 2012). The further molecular characterization of transcription factor and miRNA networks, as well as changes in epigenetic marks, will be crucial for understanding the mechanism of dedifferentiation in regenerating amphibian limbs.

Five proteins involved in canonical or non‐canonical Wnt signaling were detected in a proteomic analysis of axolotl limb blastema formation (Rao et al., 2009). These were Wnt 8, APC, the Disheveled‐binding CCDC88c, DIXDC1, and inversin. Wnt 8, APC, and DIXDC1 are part of the canonical Wnt pathway. Wnt 8 and APC were strongly upregulated, but DIXDC1, a positive regulator of the canonical pathway, was downregulated. Inversin and CCDC88c are components of the non‐canonical pathway. Inversin switches the canonical pathway to the non‐canonical pathway by targeting the Disheveled protein for degradation by the proteasome or by the activation of the c‐jun N‐terminal kinase (JNK) pathway by DVL2 and axin (Kestler & Kuhl, 2008), and CCDC88c is a negative regulator of the canonical pathway. Both were strongly upregulated. These results suggest that both canonical and non‐canonical Wnt pathways regulate blastema formation. They are consistent with the finding of Ghosh, Roy, Seguin, Bryant, and Gardiner (2008) that genes for both pathways are expressed in the regenerating axolotl limb, and with the finding that the canonical pathway (via Wnt 8) promoted zebrafish fin regeneration whereas the non‐canonical pathway was inhibitory (Stoick‐Cooper et al., 2007). The canonical Wnt pathway has also been implicated in deer antler regeneration (Mount et al., 2006) and *Xenopus* tadpole tail regeneration (Lin & Slack, 2008). Further studies will be required to understand the details of how Wnt signaling pathways regulate appendage regeneration in different species.

#### 3.3.2 Dedifferentiation of myofibers

Dismantling of phenotypic structure and function is most visible in the myofibers of regenerating adult newt limbs, but the molecular details of internal structural remodeling in dedifferentiating cells are poorly understood. Two small purine molecules dubbed myoseverin and reversine that cause cellularization of C2C12 mouse myofibers have been screened from combinatorial chemical libraries (Chen, Zhang, Wu, Schultz, & Ding, 2004; Rosania et al., 2000). Myoseverin disrupted microtubules and upregulated genes for growth factors, immunomodulatory molecules, ECM remodeling proteases, and stress‐response genes, consistent with the activation of pathways involved in wound healing and regeneration, but did not activate the whole program of myogenic dedifferentiation in newt limbs (Duckmanton, Kumar, Chang, & Brockes, 2005). Reversine treatment of mouse C2C12 myotubes resulted in mononucleate cells that mimic mesenchymal stem cells in their ability to differentiate in vitro into osteoblasts and adipocytes, as well as muscle cells (Anastasia et al., 2006). Myoseverin and reversine are thus useful in analyzing the events of structural remodeling involved in dedifferentiation and may have natural counterparts that can be isolated. Furthermore, several small molecules that inhibit GS‐3K, p38 MAP kinase, and adenylyl cyclase and activate G‐protein induce the proliferation of mononucleate mammalian C2C12 muscle cells derived by reversine treatment, thus mimicking early steps of urodele limb regeneration (Jung & Williams, 2011; Kim et al., 2012).

Recent evidence indicates that the mononucleate cells produced by fragmentation of adult myofibers involve a caspase‐induced cell death program that under other circumstances leads to apoptosis (Zitvogel, Kepp, & Kroemer, 2010), but during limb regeneration results in an autophagic program resulting in a proliferation‐competent population of myogenic cells that can redifferentiate into myofibers (Wang et al., 2015).

### 3.4 Entry into the cell cycle

[3H]‐thymidine labeling studies have shown that, as progenitor blastema cells are forming into an accumulation blastema, they enter the cell cycle and synthesize DNA. The pulse labeling index reaches 10%–30% during formation of the adult newt accumulation blastema (Loyd & Tassava, 1980; Mescher & Tassava, 1976). By contrast, the mitotic index is low, between 0.1% and 0.7% (average ∼0.4%, or 4/1000 cells) in both *Ambystoma* larvae (Kelly & Tassava, 1973) and adult newts. The total length of the cell cycle has been calculatd to be approximately 40 h for regenerating axolotl limbs (McCullough & Tassava, 1976) and 45 h for regenerating adult newt limbs (Grillo, 1971).

The fact that blastema cells synthesize DNA but divide only infrequently during formation of the accumulation blastema suggests that a large proportion of dedifferentiating cells arrest in G2 (Mescher & Tassava, 1976). Further indirect evidence for G2 arrest is the strong upregulation of the ecotropic viral integration factor 5 (Evi5) throughout blastema formation in regenerating axolotl limbs and regenerating ear hole tissue of MRL/mpj mice (Heber‐Katz et al., 2013; Rao et al., 2009). Evi5 is a centrosomal protein that accumulates in the nucleus during early G_1_ in mammalian cells and prevents them from prematurely entering mitosis by stabilizing Emi1, a protein that inhibits cyclin A degradation by the anaphase‐promoting complex/cyclosome (APC/C) (Eldridge et al., 2006). At G2, Emi1 and Evi5 are phosphorylated by Polo‐like kinase 1 (PLK1) and targeted for ubiquitin‐driven degradation, allowing the cell to enter mitosis. Thus, high levels of Evi5 during blastema formation may restrain cells from entering mitosis until they are fully dedifferentiated and present in enough numbers to form an accumulation blastema (Rao et al., 2009). To test this hypothesis, it will first be necessary to determine the spatiotemporal expression pattern of Emi1 and Evi5. The hypothesis predicts that these proteins would be expressed at high levels in both migrating wound epidermis (which does not divide) and the mesenchyme of the accumulation blastema, and that expression would decrease as the cells transit to a normal cell cycle during blastema growth.

The signals that induce liberated cells to enter the cell cycle have been studied in detail in myotubes derived from the newt A1 cell line of myogenic precursors (Ferretti & Brockes, 1988). Serum stimulation of A1 myotubes induces their partial dedifferentiation, as manifested by downregulation of the *Myf5* gene (Imokawa, Simon, & Brockes, 2004). A thrombin‐activated factor present in the serum of all vertebrates tested thus far (Straube, Brockes, Dreschel & Tanaka, 2004; Tanaka, Gann, Gates, & Brockes, 1997) promotes progression through G_1_ and S in cultured newt myotubes by activating a sustained extracellular signal‐regulated kinase (ERK1/2) pathway that downregulates the Sox6 and p53 (tumor suppressor) proteins (Yun, Gates, & Brockes, 2013, 2014), facilitating phosphorylation and inactivation of the retinoblastoma protein (pRb) to block entry into S‐phase. Mouse myonuclei do not synthesize DNA in response to serum stimulation (Tanaka et al., 1997). Newt blastema extract promotes dedifferentiation and DNA synthesis in both newt and mouse C2C12 myotubes in vitro (McGann, Odelberg, & Keating, 2001), and mouse myonuclei will synthesize DNA if they are part of a mouse/newt heterokaryon (Velloso, Simon, & Brockes, 2001). Yun et al. (2014) have shown that mouse myotubes briefly activate the ERK1/2 pathway, but do not sustain the activity, and thus fail to deactivate pRb. In addition, mammalian myotubes must overcome an additional block to DNA synthesis by the ARF tumor suppressor protein encoded by the *ink4a* locus, which is expressed only in taxa above the urodeles (Pajcini, Corbel, Sage, Pomerantz, & Blau, 2010).

Although the thrombin‐activated protein is both necessary and sufficient to stimulate the entry of myonuclei into the cell cycle, it is not sufficient to drive them through mitosis, and they arrest in G_2_. Myofiber cellularization and cell cycle entry are independent of one another, since cell‐cycle‐inhibited myofibers implanted into newt limb blastemas break up into mononucleate cells (Velloso, Kumar, Tanaka, & Brockes, 2000). Mitosis, however, requires mononucleate cell status. The identity of the thrombin‐activated protein is unknown, although some evidence suggests that it may be a potent growth factor required in very small amounts (Straube et al., 2004). Sugiura, Wang, Barsacchi, Simon, and Tanaka (2016) reported that a MARCKS (myristoylated alanine‐rich C‐kinase substrate)‐like protein called the muscle LIM protein (MLP) initiates entry into the cell cycle of muscle‐derived blastema cells. This protein clusters phylogenetically with other vertebrate MLPs, which generally play a role in muscle differentiation. MLP is secreted within 12 h after amputation of an adult newt limb and its activity is essential for entry into the cell cycle. Whether MLP is a general initiator of cell cycle entry for blastema cells derived from other limb tissues or larval limbs is unknown, as is its relation to the thrombin‐activated factor.

### 3.5 Molecular markers of blastema cells

In addition to the antigens expressed by the AEC, several antigens specific to mesenchymal blastema cells have been identified by immunochemical methods. The antigen 22/18 is expressed by 80% of newt medium bud blastema cells, in cultured newt blastema cells, and during the tissue regeneration of newt muscle (Ferretti & Brockes, 1988; Griffin, Fekete, & Carlson, 1987; Kintner & Brockes, 1984, 1985). This antigen is an intermediate filament that undergoes a conformational change during limb regeneration (Ferretti & Brockes, 1990). Its expression appears to be nerve‐dependent, because it is not expressed in the limb bud or in regenerating aneurogenic limbs (Fekete & Brockes, 1988; Ferretti & Brockes, 1991; Gordon & Brockes, 1988). Three keratins, keratins 8 and 18 and a newt type II keratin, NvKII, are expressed in newt blastema cells (Ferretti, Brockes, & Brown, 1991; Ferretti, Fekete, Patterson, & Lane, 1989). NvKII and 9G1 are also expressed in the AEC of the newt limb blastema. The functions of these proteins are unknown. The gene encoding the PRRX1 paired homeobox protein is expressed in the nuclei of axolotl and *Xenopus* limb blastema cells (Satoh, Gardiner, Bryant, & Endo, 2007; Suzuki, Satoh, Ide, & Tamura, 2007). This protein is essential for limb bud skeletal patterning (Nohno et al., 1993). It is activated by dermal fibroblasts during blastema formation in the amputated axolotl limb and its expression is induced by MMP activity (Satoh, Makanae, Hirata, & Satou, 2011).

### 3.6 Tissue contributions to the blastema

**Figure 2:**
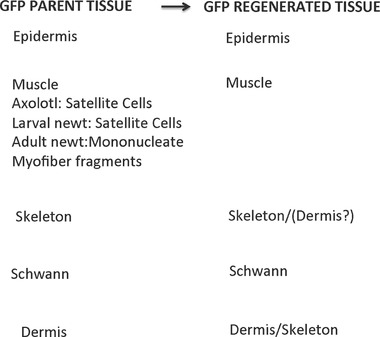
Blastema cells have a memory of cellular origin. Results of experiments tracing tissues grafted from transgenic GFP axolotls to white axolotls, based on data from Kragl et al. (2009), Sandoval‐Guzman et al. (2014) and Tanaka et al. (2016). Blastema cells give rise to the same tissue of origin in the regenerate, with the exception of dermal cells, which can also transdifferentiate to skeletal cells. Muscle in larval axolotls and newts is regenerated by satellite cells, whereas regenerated muscle in adult newts is derived primarily from mononucleate cells produced by fragmentation of cut myofibers

Extensive histological and experimental analysis has shown that blastema cells originate from the mesodermal tissues directly subjacent to the wound epidermis (Butler & O'Brien, 1942; Thornton, 1968). The wound epidermis itself makes no contribution to this cell population (Riddiford, 1960). Nearly half the cells of the blastema are derived from dermal fibroblasts (Muneoka, Fox, and Bryant, 1986a), but the total fibroblast contribution is probably well above 50% when the fibroblasts of the periosteum, muscle interstitial tissue, and nerve sheath are considered. Experiments in which transgenic green fluorescent protein (GFP) neurula stage axolotl tissues contributing to the limb were grafted in place of their counterparts in non‐GFP neurulae and the developed limbs amputated showed that dermal fibroblasts, Schwann cells, skeletal cells, and myogenic cells contribute to the blastema (Kragl et al., 2009; Fig. 2).

The myogenic contribution, however, varies with species and phase of the life cycle. Satellite cells are the source of regenerated muscle in larval and metamorphosed axolotls (Sandoval‐Guzman et al., 2014). The larval newt limb also mobilizes satellite cells to regenerate muscle, but the adult newt limb switches to dedifferentiation of mononucleate myofiber fragments as the primary source of muscle progenitors (Tanaka et al., 2016; Young, Bailey, Markwald, & Dalley, 1985). Nevertheless, satellite cells have been identified by electron microscopy in adult newt limb muscle (Cameron, Hilgers, & Hinterberger, 1986) and can form myotubes when explants of this muscle are cultured in vitro (Schrag & Cameron, 1983). These cells may be involved in the regeneration of injured muscle and in the repair of muscle in the overlap region between the blastema and disorganized stump tissues in regenerating adult newt limbs. Why there should be dual mechanisms for muscle regeneration after muscle injury versus amputation is an interesting question that has not been sufficiently explored.

There are several questions yet to be answered about the origin of the blastema. For example, what percentage of the blastema cells is contributed by non‐dermal fibroblasts? Might specific subpopulations of progenitor cells exist in dermal and other fibroblast populations that contribute to the blastema as opposed to dedifferentiation? Is the switch from satellite cells to myofiber dedifferentiation in adult newt limb regeneration an all or none event, or is it gradual, and what regulates this switch? Do chondrocytes contribute to the blastema? Hay (1958) described the dedifferentiation of chondrocytes in the regenerating larval urodele limb, and Onda and Tassava (1991) described the strong expression of an antigen, 9G1, in dedifferentiating newt limb chondrocytes during histolysis and blastema formation. However, triploid‐labeled cartilage gave rise to few chondrocytes in the regenerate when grafted to the diploid axolotl limb (Muneoka, Fox et al., 1986a; Steen, 1968) and chondrocytes were not observed to contribute to the blastema at all in another set of experiments where GFP‐labeled cartilage was injured in evoking the formation of a supernumerary blastema and limb (McCusker, Diaz‐Castillo, Sosnik, & Gardiner, 2016). This issue should be explored further to determine whether the skeletal contribution to the blastema is via the periosteum, cartilage/bone, or both, and whether there may be species and developmental stage related differences in skeletal contribution to the blastema, as for muscle.

### 3.7 Blastema cells have lineage‐specific and positional memories

Experiments grafting tissues and cells from animals transgenic for the GFP have shown that blastema cells have two types of cellular memory. The first is a lineage‐specific memory of limb and parent cell phenotype (Kragl et al., 2009). This memory dictates that blastema cells derived from muscle and Schwann cells redifferentiate in a lineage‐specific manner as myogenic cells and Schwann cells (Fig. 2). Blastema cells derived from fibroblasts differentiate into fibroblasts, but have more flexibility in being able to transdifferentiate into chondrocytes and tendon cells. In fact, a complete skeleton can regenerate distal to the plane of amputation from dermal fibroblasts of the skin, as first shown in experiments amputating boneless limbs of newts (Bischler & Guyenot, 1925; Weiss, 1925) and later by Namenwirth (1974) in experiments grafting normal skin in place of the skin of irradiated axolotl limbs.

The second type of memory is positional identity, a memory of the position of origin of blastema cells in relation to their neighbors (Mittenthal, 1981). Positional memory is restricted to fibroblast‐derived blastema cells, and is the basis of the rule of distal transformation, ensuring that only the missing distal structures are regenerated (Nacu et al., 2013). Blastema cells derived from muscle and Schwann cells lack a memory of their position of origin. The position they come to occupy during pattern formation is flexible and regulated by fibroblast‐derived blastema cells. Phenotypic and positional memory is probably due to retention of a major part of the original epigenetic codes imposed on the genome in developing limb buds, as reflected in a stably maintained histone methylation pattern of blastema cell DNA (Hayashi et al., 2015).

**Figure 3:**
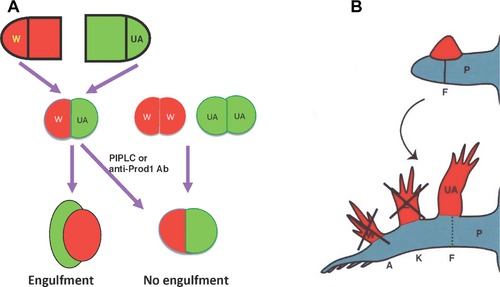
Positional memory is encoded in the cell surface. (A) Blastemas derived from the same level (W/W, UA/UA) fuse in a straight line when juxtaposed in culture, but when derived from different levels (W/UA), the more proximal blastema engulfs the distal one. Engulfment is prevented by treating the culture with PIPLC or an antibody to Prod1. (B) Medium bud blastemas derived from the wrist, elbow, and mid upper arm (red) grafted to the dorsal surface of the blastema/stump junction of a hindlimb regenerating from the mid‐femur sort to their corresponding levels of ankle (A), knee (K), and mid‐femur (F) of the regenerating hindlimb. Retinoic acid, which proximalizes the positional identity of blastema cells, abolishes the distal sorting of the wrist and elbow blastemas, so that they behave like upper arm blastema. P, posterior; K, knee; A, ankle; UA, upper arm

Positional identity is encoded in the blastema cell surface, as shown by in vitro and in vivo assays (Fig. 3). When pairs of proximal and distal blastemas were juxtaposed at their bases and cultured in hanging drops, the proximal blastema engulfed the distal one, whereas a pair of blastemas from the same level simply fused in a straight line (Nardi & Stocum, 1983). Based on the work of Steinberg (1978), this result suggests a distal (stronger) to proximal (weaker) gradient of blastema cell intercellular adhesion. The existence of this gradient in vivo was demonstrated by an “affinophoresis” assay in which undifferentiated blastemas from wrist, elbow, and mid upper arm levels of the forelimb were grafted individually to the blastema‐stump junction of hindlimbs regenerating from the mid‐femur. The wrist and elbow blastemas sorted to their corresponding levels on the regenerating host blastema (ankle and knee, respectively) while the mid upper arm blastema remained at the mid‐femur level (Crawford & Stocum, 1988a; Egar, 1993). Further evidence that positional identity is encoded in cell surface adhesion molecules was obtained by showing that retinoic acid (RA), which proximalizes the positional identity of axolotl limb blastema cells **(**Maden, 1982a), abolished the distal sorting of blastemas in the affinophoresis assay (Crawford & Stocum, 1988b).

The sorting behavior of distal from proximal cells was confirmed by experiments grafting clusters of marked cells from an early wrist blastema into the prospective humeral mesenchyme of an early mid upper arm blastema, where they sorted out to participate in hand formation (Echeverri & Tanaka, 2005). Genetic marking experiments showed that the PD adhesive differentials exist at the single cell level (Kragl et al., 2009). Position‐dependent adhesion of cells in the developing and regenerating limb bud of the early *Xenopus* tadpole was also demonstrated by the sorting out of distal and proximal cells from an initial mixture of the two (Ohgo et al., 2010).

A cell surface molecule implicated in establishing PD adhesive differentials and coordination of proliferation and patterning in the regenerating newt limb is Prod1, a member of the Ly6 family of three‐finger proteins that is anchored to the cell surface by a glycosylphosphatidylinositol linkage (Morais da Silva, Gates, & Brockes, 2002). Antibody blocking of Prod1 or its removal from the blastema cell surface by phosphatidylinositol‐specific phospholipase C (PIPLC) inhibited the recognition of adhesive differentials between distal and proximal blastemas (Fig. 3), whereas overexpression of Prod1 in distal blastema cells caused them to sort to a more proximal (less adhesive) position when grafted into proximal blastemas (Echeverri & Tanaka, 2005). Other surface molecules that may be involved in position‐dependent adhesion of blastema cells are CD59, ephrins, and cadherins. Antibodies to CD59, which is expressed in a high to low gradient along the PD axis of the gecko tail, abolished the normal engulfment of distal tail blastemas by proximal blastemas in vitro (Wang et al., 2011). Antibodies to the EphA4 receptor and to N‐cadherin, or cleaving of ephrin A ligands from the cell surface with phospholipase C, abolished the sorting of proximal and distal chick limb bud cells from one another (Wada, 2011; Wada, Kimura, Tanaka, Ide, & Nohno, 1998; Yajima, Yonei‐Tamura, Watanabe, Tamura, & Ide, 1999), and RA treatment prevented the sorting of distal cells of the chick limb bud from proximal cells (Tamura, Yokoyuchi, Kuroiwa, & Ide, 1997). There is a need for a more refined analysis of position‐dependent cell surface molecular signatures if we are to understand how proliferation and patterning are integrated. It is also possible that positional information may reside in the ECM surrounding fibroblast‐derived blastema cells (Phan et al., 2015).

### 3.8 Macrophages play an important role in blastema formation

The immune system plays an important role in wound repair and regeneration (for reviews see Eming, Wynn, & Martin, 2017; Mescher 2017; Mescher, Neff & King, 2017). Macrophages of the innate immune system are a central mediator of wound repair in mammals. The macrophages entering the wound are initially pro‐inflammatory in line with their bactericidal and phagocytic activities and their secretion of growth factors and cytokines that regulate inflammation. The macrophages then change to an anti‐inflammatory phenotype secreting factors that resolve inflammation and initiate structural repair by fibroblasts when activated by interleukin 4/13 (IL‐4/13). IL‐4/13‐dependent macrophage proliferation is enhanced by their production of defense collagens (Minutti et al., 2017), and the types of defense collagens produced are dictated by the tissue location of the macrophages. Making this pro‐inflammatory to anti‐inflammatory switch requires that the macrophages first sense apoptotic neutrophils (Bosurgi et al., 2017). The result is “normal” scar formation, but not regeneration.

A positive correlation between tissue damage, cell senescence, and the ease of in vivo reprogramming of somatic cells by Yamanaka transcripton factors (OSKM) has been reported (Mosterio et al., 2016). The cells of tissues lacking p16^INK4a^/ARF or treated with the senescence inhibitor navitoclax exhibit reduced or no senescence and their ability to be reprogrammed is compromised, whereas injured tissues that lack the p53 protein (guardian of the genome that eliminates aberrant cells with DNA damage) have large numbers of senescent cells that accumulate in the tissue, produce elevated amounts of IL‐6, and are more receptive to reprogramming. These results were reproducible in vivo by culturing cells either with senescent cells or in medium conditioned by senescent cells.

Cell senescence thus seems to be essential to mammalian wound repair, cellular reprogramming, and most likely to signal stem cells to proliferate and replace senescent and apoptotic cells. The downside is that senescent cells accumulate in mammalian tissues with age, reflecting a process that decreases the ability to regenerate worn out cells. Thus, potential therapies for age related loss of tissue regenerative ability might involve interventions that eliminate accumulation of senescent cells. In fact, drug‐induced elimination of senescent cells in mice was found to retard organ deterioration and tumor formation and to extend life span by 20% (Baker et al., 2016). Likewise, elimination of the senescent foamy macrophages associated with atherosclerosis reduced plaque formation in atherosclerosis‐prone mice by 60% (Childs et al., 2016).

The innate immune system has long been postulated as a major factor that determines whether appendages can regenerate or not (Harty, Neff, King, & Mescher, 2003; Mescher & Neff, 2006; Mescher, Neff, & King, 2013; for reviews). Urodeles, which can regenerate limbs as larvae and adults, have a much less developed immune system than anurans (frogs and toads), which regenerate limbs only as early tadpoles, and mammals, which have no limb regenerative power except for digit tips. Macrophages are particularly important for the events of blastema formation during urodele limb regeneration (Godwin & Brockes, 2006; Godwin & Rosenthal, 2014; Mescher, 2017; Mescher et al., 2017; for reviews).

Godwin et al. (2013) demonstrated that pro‐ and anti‐inflammatory cytokines are upregulated during blastema formation in regenerating axolotl limbs, coincident with a significant enrichment of macrophages, which produce MMPs and make the pro‐ to anti‐inflammatory switch, just as in mammalian wound repair. Macrophage depletion by liposome‐encapsulated clodronate during blastema formation results in regenerative failure and scarring of the limb stump. The epidermis closes the wound, but does not develop an AEC because dermal scar tissue is interposed between the wound epidermis and underlying tissues. By contrast, depletion after a blastema enters the growth phase only delays regeneration. These results suggest a central role for macrophages in limb regeneration by resolving inflammation by shifting cytokine ratios in favor of the anti‐inflammatory subset, and by ECM degradation, including the basement membrane. Macrophages are also necessary for the regeneration of ear punch hole tissue in the African spiny mouse *Acomys*, which occurs by the formation of a blastema around the rim of the wound (Simkin, Gawriluk, Gensel, & Seifert, 2017). Pro‐inflammatory macrophages fail to penetrate the blastema tissue, but clodronate treatment eliminating both pro‐ and anti‐inflammatory macrophages results in scarring.

A major role of macrophages during limb regeneration is to remove senescent cells. Very few senescent or apoptotic cells are detected in regenerating urodele limbs (Mescher, White, & Brokaw, 2000). Yun, Davaapil, and Brockes (2015) demonstrated that cell senescence is induced during blastema formation in amputated axolotl limbs but that senescent cells do not accumulate because they are cleared by macrophages. Furthermore, this clearance is obligatory for blastema formation. This may not be true for all vertebrates that regenerate appendages; macrophages and neutrophils were dispensable for regeneration of zebrafish fins (Mathew et al., 2007). The question then arises whether other macrophage functions in limb regeneration require the engulfment of senescent cells, or whether senescent cells release factors that facilitate reprogramming of limb cells to blastema cells prior to being eliminated by macrophages? Other questions revolve around the role of adaptive immune cells in limb regeneration, and the nature of the immune environment of mouse digit tips that allows them to regenerate.

### 3.9 Blastema cell migration and accumulation

The G_2_ arrest of blastema cells indicates that the blastema forms exclusively by migration and aggregation of cells beneath the AEC rather than mitosis. The AEC appears to direct this process, as shown by experiments in which shifting the position of the AEC laterally caused a corresponding shift in blastema cell accumulation, and transplantation of an additional AEC to the base of the blastema resulted in supernumerary blastema formation (Thornton, 1960a; Thornton & Thornton, 1965). Guidance by nerves was ruled out, since similar experiments on aneurogenic limbs also resulted in eccentric blastema formation (Thornton & Steen, 1962). Directional migration is provided by transforming growth factor β1 (TGF‐β1) stimulated fibronectin produced by basal cells of the AEC (Christensen & Tassava, 2000). Inhibition of TGF‐β1 expression by the inhibitor of SMAD phosphorylation, SB‐431542, reduces fibronectin expression, resulting in failure of blastema formation (Levesque et al., 2007).

## 4 BLASTEMA GROWTH

Growth of the accumulation blastema requires two synergistic inputs to break G_2_ arrest and divide. First is the expression of mitosis‐promoting factors by regenerating nerve axons and the AEC. Second is the interaction of blastema cells with non‐neighboring anterior−posterior (AP) or dorsal−ventral (DV) positional identities. Unless these two conditions, along with re‐vascularization, are met, dedifferentiating cells may accumulate, but fail to persist and divide, and disappear.

### 4.1 Role of the nerve in blastema growth

Nerves have long been recognized as the electrical system of the body, but their role in niche support for stem cells of various organs, for regulation of wound repair, and for cell proliferation in amphibian limb regeneration is of more recent recognition (Kumar & Brockes, 2012; Pirotte, Leynen, Artois, & Smeets, 2015).

The English physician Tweedy John Todd reported in 1823 that newt hindlimbs failed to regenerate if the sciatic nerve was severed at the time of amputation. A hundred years later Schotte (1926) and Locatelli (1929) confirmed a neural requirement for limb regeneration. Butler and Schotte (1941) and Schotte and Butler (1941) showed that larval salamander limbs denervated at any time between amputation and the medium bud stage regressed to the level of the shoulder and formed a scar. Compression injury or skeletal fracture of denervated larval limbs without amputation also resulted in limb regression distal and proximal to the injury (Thornton, 1954). Denervated and amputated adult newt limbs do not regress, but simply scar at the level of amputation (Singer & Craven, 1948). Once re‐innervation occurs, re‐opening the wound to remove scar tissue allows both larval and adult limbs to regenerate. Regression of denervated larval limbs is due to injury‐activated proteolytic enzymes such as MMPs and can be prevented by grafting a medium bud blastema to the amputation surface (Schotte & Harland, 1943; Schotte, Butler, & Hood, 1941), most probably due to the synthesis of TIMPs by the blastema, although this has not been demonstrated directly. Whether or not the nerve has any influence on TIMP expression is unknown.

Marcus Singer carried out a comprehensive series of studies on the role of the brachial nerves (spinal nerves 3, 4, and 5) in regeneration of the adult newt forelimb (Singer, 1942, 1943, 1945, 1946a, b, 1947a, b; Singer & Egloff, 1949) which revealed that a threshold number of axons (later expressed as amount of axoplasm per unit area of newt limb tissue) is required for regeneration, and that the threshold is different at different PD levels of the limb. The results of these studies were synthesized into the neurotrophic hypothesis (Singer, 1952, 1964, 1965), which states that the nerves provide a threshold level of trophic factors essential for the survival and proliferation of blastema cells. Singer (1943, 1945) and Sidman and Singer (1960) found that, although augmentation of the motor nerve supply in the absence of sensory innervation can support regeneration, under normal circumstaces only the sensory innervation is capable of meeting the threshold requirement; the normal motor and sympathetic innervations cannot.

Later molecular studies showed that denervation does not affect the DNA polymerase activity or enzymes that catalyze synthesis of nucleotide precursors (Dresden & Moses, 1973; Manson, Tassava, & Nishikawara, 1976), but drastically reduces the transcription of all classes of RNA (Bantle & Tassava, 1974; Kelly & Tassava, 1973; Morzlock & Stocum, 1972) for a reduction in total RNA synthesis of 75% (Dresden, 1969). Expression of genes specific to wound repair or muscle did not differ in amputated control and denervated limbs, but the transcription of genes associated with proliferation was reduced in denervated limbs coincident with the beginning of the growth phase (Monaghan et al., 2009).

Denervation reduces protein synthesis by 50%−70% via reduction in transcription without any effect on the amino acid precursor pool, rate of protein degradation, or rate of translation (Choo, Logan, & Rathbone, 1978; Dresden, 1969; Lebowitz & Singer, 1970). Neural and hormonal input to cultured adult newt limb blastemas maintains DNA and protein synthesis by the blastema cells (Vethamany‐Globus, Globus, & Tomlinson, 1978). The protein profile changes throughout blastema growth and differentiation (Dearlove & Stocum, 1974; Singer, 1978; Singer & Ilan, 1977; Tsonis, Mescher, & Del‐Rio Tsonis, 1992). Changes in protein synthesis are reflected in the ECM, particularly in the synthesis of proteoglycan and collagen‐associated glycosaminoglycans. Hyaluronate is the major glycosaminoglycan synthesized during blastema formation in adult newt limbs (Smith, Toole, & Gross, 1975) and is reduced the most by denervation (Mescher & Munaim, 1986; Young, Dalley, & Markwald, 1989). Consistent with the scarring of denervated and amputated newt limbs, collagen fibrillogenesis begins prematurely (Bryant, Fyfe, & Singer, 1971; Vanrapenbush & Lasalle, 1989). The effect of denervation on a wide array of genes and proteins revealed by global genomic and proteomic analysis (Looso et al., 2013; Monaghan et al., 2009; Rao et al., 2009, 2014; Voss et al., 2015) is now wide open for investigation.

Blastemas that have achieved the medium bud stage become independent of the nerve for morphogenesis and differentiation. Their cells remain nerve‐dependent for proliferation, however, and form miniature regenerates when denervated (Maden, 1981; Powell, 1969; Schotte & Butler, 1944; Singer & Craven, 1948). The mitotic index of the blastema is reduced to zero by denervation at any stage of blastema growth (Goldhamer & Tassava, 1987; Maden, 1978b; Tassava, Bennett, & Zitnik, 1974).

The relationship between regenerating nerve fibers and blastema cells is a reciprocal one. The regeneration of nerve fibers into the blastema is dependent on factors produced by the blastema cells. Regeneration of axons from nerve cell bodies is promoted in vitro by co‐culture of neurons with blastema tissue (Richmond & Pollack, 1983). Several known neurotrophic factors such as brain‐derived neurotrophic factor, neurotrophins 3 and 4, glial‐derived neurotrophic factor, and hepatocyte growth factor/scatter factor can substitute for blastema tissue in promoting axon outgrowth in vitro (Tonge & Leclere, 2000). These factors are the ones produced by Schwann cells that promote neuron survival and axon outgrowth in regenerating peripheral nerves of mammals, raising the question of whether they might be produced by the subpopulation of blastema cells derived from Schwann cells. Regardless, axon outgrowth is significantly more vigorous with blastema tissue, suggesting that blastema cells produce other, as yet unidentified, factors that encourage neuron survival and axon outgrowth. A comparison of genes expressed by axolotl dorsal root ganglia (DRG) cells in the presence and absence of blastema cells revealed 27 DRG genes that were differentially expressed in the presence of blastema cells (Athippozhy, Lehtberg, Monaghan, Gardiner, & Voss, 2014).

### 4.2 Role of the AEC in blastema growth

The AEC is equally important for limb regeneration. Transplanting whole skin over the amputation surface (Chew & Cameron, 1983; Mescher, 1976), or inserting the ends of amputated limbs or regenerates into a pocket made under flank skin (Butler, 1955; Polezhaev & Faworina, 1935) or into the coelom (Deck, 1955; Goss, 1956a, b) results in lack of blastema formation. Thornton (1954) noted that the AEC is always present during blastema growth and patterning and fails to form in anuran late tadpole limb buds that have lost the ability to regenerate (Thornton, 1956). Regeneration fails in amputated larval urodele limbs when the AEC is repeatedly removed (Thornton, 1957) or its formation is suppressed by UV irradiation (Thornton, 1958). Contact of the AEC with subjacent blastema cells is crucial for blastema cell proliferation; interposition of dermis or the formation of basement membrane between the two inhibits regeneration (Chew & Cameron, 1983; Kim & Stocum, 1986b; Stocum & Crawford, 1987).

Depriving the growing blastema of the AEC has both similar and different effects to denervation. Blastema mesenchyme stripped of its epidermis by chelation and implanted into a dorsal fin tunnel such that it cannot contact epidermis forms a miniature regenerate but, unlike denervated blastemas, one that is truncated distally (Stocum & Dearlove, 1972). Positioning the mesenchyme so that its distal tip protrudes from the tunnel and is re‐covered by fin epidermis also produces a miniature regenerate, but one that is complete in the PD axis, as in denervated limbs. In these experiments, the blastema mesenchyme is also denervated, but has the opportunity to receive innervation by nerves of the dorsal fin. Cell proliferation was not directly assessed, but miniaturization suggests that the AEC plays a role in mitosis. Distal truncation suggests that it also has a role in PD patterning, but it is possible that removal of the AEC kills the distal‐most blastema cells, as has been shown for the chick limb bud (Dudley, Ros, & Tabin, 2002; Rowe & Fallon, 1982). Direct evidence for a mitogenic role of the AEC is that DNA synthesis and mitosis of AEC‐free blastemal mesenchymes cultured in vitro transfilter to dorsal root ganglia or brain neurons are reduced by a factor of 3−4 (Globus, Vethamany‐Globus, & Lee, 1980; Smith & Globus, 1989). The molecular effects of AEC deprivation on subjacent blastema cells have not been assessed, but 125 genes that are highly upregulated in the AEC have been identified by transcript analysis (Campbell et al., 2011).

### 4.3 The functional relationship of nerve and AEC: hypotheses

The fact that limb regeneration is dependent on both nerves and the AEC suggests a functional relationship between the two. Motor axons make intimate contact with blastema cells that are probably myogenic (Lentz, 1967). Singer (1949), Thornton (1954, 1956) and Salpeter (1965) observed that the AEC was richly innervated by regenerated sensory axons leading Thornton (1954) to propose that sensory innervation induces formation of the AEC. This idea was questioned by Singer, however, because augmenting the number of motor axons could support regeneration in the absence of sensory nerves (Sidman & Singer, 1960). Augmentation of motor axons was achieved by cutting brachial nerves 3, 4, and 5, ablating the spinal ganglia, and connecting the cut ends of the nerves to their ventral roots, allowing motor regeneration through the empty sensory endoneurial tubes of spinal nerves 3, 4, and 5. The regenerated motor nerves were randomly distributed throughout the blastema and did not enter the wound epidermis. Nevertheless, the AEC formed and was maintained, and regeneration took place normally, confirming that the effect of the nerve on regeneration was quantitative rather than qualitative. Thornton (1960b) repeated this experiment on larval *Ambystoma* limbs with the same result, leading to the conclusion that there was no interdependent functional relationship between nerves and AEC for regeneration (Singer, 1965). The function of wound epidermis and AEC was considered to be removal of tissue debris and provision of external secretions. Later, Endo, Bryant, and Gardiner (2004) found that an AEC formed independently of the nerve after creating a wound on an axolotl limb, but regressed unless it became innervated, indicating that Thornton was at least partly correct in his view that there is some sort of dependence of the AEC on innervation.

Three major ideas have been put forward about the nature of the nerve:AEC functional relationship in promoting blastema cell proliferation. These are (1) the nerve and AEC provide separate factors with different roles in the cell cycle; (2) the AEC provides all factors necessary for the cell cycle but is nerve‐dependent to express them; (3) the nerve and AEC express the same mitogen that drives blastema growth. In all three hypotheses, the effect of the nerve is quantitative, as found by Singer (1952).

### 4.4 Nerve and AEC have separate roles in the cell cycle

In the 1970s, Roy Tassava and his students conducted a broad ranging analysis of DNA synthesis and mitosis by blastema cells that suggested separate but synergistic roles of nerves and AEC in regeneration at the level of the cell cycle (Tassava & McCullough, 1978). Labeling of amputated limbs deprived of nerves or wound epidermis with [3H]‐thymidine showed that DNA synthesis of nascent blastema cells is independent of both these tissues, but that in the absence of either one the labeled cells were arrested in G_2_ of the cell cycle (Kelly & Tassava, 1973; Mescher, 1976; Tassava et al., 1974). The cells can be rescued by re‐innervation (Olsen, Barger, & Tassava, 1984), but otherwise undergo apoptosis and are removed by macrophages (Mescher et al., 2000; Yun et al., 2015). Coincident with re‐innervation of the AEC, the labeling and mitotic indices of the accumulation blastema rise as much as 10‐fold (Mescher & Tassava, 1976; Loyd & Tassava, 1980). These increases do not take place in limbs that are either denervated or deprived of wound epidermis. [3H]‐thymidine pulse labeling studies indicate that the final cycling fraction of blastema cells is 92%−96% in the regenerating limbs of axolotl larvae and over 90% in those of adult newts (Goldhamer & Tassava, 1987; Tomlinson, Goldhamer, Barger, & Tassava, 1985).

Based on these results, Tassava and Mescher (1975) proposed the hypothesis that injury stimulates blastema cells to enter the cell cycle and that the AEC maintains the cells in an undifferentiated state that keeps them in the cell cycle and renders them responsive to mitogenic signals supplied by the nerve. This idea is consistent with the results of in vitro transfilter experiments by Globus et al. (1980) and Smith and Globus (1989), demonstrating that adult newt blastema cells grown opposite dorsal root ganglia or brain cells fail to undergo mitosis in the absence of the wound epidermis, withdraw from the cell cycle, and differentiate as cartilage, whereas in the presence of epidermal cells and neural tissue they are maintained in an undifferentiated state and proliferate.

### 4.5 The AEC is dependent on the nerve to express blastema cell mitogens

In this hypothesis (Stocum, 2011) the AEC provides the mitogenic factor(s) for proliferation but requires neurotrophic factor(s) to express them. This hypothesis is derived from the results of experiments on limb bud development, aneurogenic limb regeneration, and the rescue of denervated limbs by neurotrophic and AEC factors.

A reciprocal epithelial:mesenchymal interaction promotes the growth of amniote embryonic limb buds (Saunders, 1948; Zwilling & Hansborough, 1956). Briefly, the mesenchyme expresses Fgf10, which induces and maintains the AER, and the AER expresses Fgf8, which maintains Fgf10 expression and proliferation of the subjacent mesenchyme cells (see Gilbert & Barresi, 2016, for a review). Although an AER is not present as a morphological entity in embryonic amphibian limb buds (Sturdee & Connock, 1975), the apical ectoderm/epidermis has the same outgrowth‐promoting function (Balinsky, 1935; Steiner, 1928; Tarin & Sturdee, 1971; Tschumi, 1957). The apical epidermis of amputated amphibian limb buds, which regenerate readily, is configured into a visible AEC. Yokoyama et al. (2000) demonstrated that mesenchymal Fgf10 maintains Fgf8 expression by the AEC in regenerating *Xenopus* limb buds, and vice versa. *Xenopus* limbs lose the power of regeneration as they differentiate and form a blastema of fibroblast‐like cells (Dent, 1962; Van Stone, 1964). This loss is accompanied by a loss of Fgf10 expression by the fibroblastema and loss of Fgf8 expression by the AEC (Yokoyama et al., 2000) due to changes in the limb bud cells related to their differentiation (Filoni, Bernardini, & Cannata, 1991; Sessions & Bryant, 1988). Fgf10‐soaked beads placed on the amputation surface of regeneration‐deficient limbs of *Xenopus* late tadpoles restore Fgf8 expression in the AEC and digit regeneration, although not more proximal structures (Yokoyama, Ide, & Tamura, 2001).

The neural requirement for regeneration is imposed on the developing limb bud only as it becomes innervated at late stages (Fekete & Brockes, 1987). Urodele limb buds rendered aneurogenic by extirpating the neural tube during embryogenesis never acquire nerve dependence for regeneration (Yntema, 1959a, b), but presumably remain dependent on the AEC for blastema cell mitosis. Steen and Thornton (1963) found that sleeves of aneurogenic limb skin of young larvae packed with [^3^H]‐thymidine‐labeled internal tissues of neurogenic larval limbs regenerated, whereas regeneration failed when the skin of aneurogenic limbs was replaced with skin from innervated limbs. These results suggest that innervation does not alter the requirement of blastema cells for mitogens, but rather decreases the capacity of the AEC to provide them. While it could be argued that the results reflect dermal aneurogenic versus neurogenic contributions of blastema cells, young larval limbs do not have a well‐developed dermis (Stearner, 1946), and the blastemas derived from the aneurogenic skin/neurogenic internal tissues combination contained many labeled cells, indicating their origin from the transplanted musculoskeletal tissue.

Nerve dependence/independence can be oscillated back and forth. Nerve dependence of aneurogenic larval limbs can be instituted by transplanting them to neurogenic larvae. If the limbs are then denervated for a period of time, they can regain nerve independence (Thornton & Thornton, 1970). Even adult newt limbs showed some capacity for nerve‐independent regeneration when maintained in a denervated condition after grafting them to the back (Singer & Mutterperl, 1963). Singer (1965) explained the ability of limb buds and differentiated aneurogenic limbs to regenerate by postulating that all their tissues have the capacity to produce the neurotrophic factor. Production of the factor by these tissues is suppressed as the limb becomes innervated, but in some cases can be restored under conditions of denervation.

Reasoning from these facts, a model to explain blastema cell proliferation in both aneurogenic and neurogenic limbs is that the AEC provides diffusible mitogens, but the expression of these mitogens becomes dependent on neural factors supplied by the sensory axons innervating the AEC as the limb differentiates. If this model is correct, we should be able to define the AEC and nerve factors involved in this interaction. Candidates for these roles should meet several minimal criteria (Brockes, 1984). First, they should be expressed by the AEC or DRG cell bodies innervating the limb. Further criteria to be an AEC mitogen are expression of the mitogen's receptor in the blastema mesenchyme, loss of mitogen expression by denervation, ability of the mitogen to support regeneration of denervated or AEC‐deprived limbs from early blastema formation to digit stages, and expression of the mitogen by the AEC of regenerating aneurogenic limbs. Neural factors should be transported from DRG cell bodies along limb sensory nerve axons to the AEC where they bind to their receptor, denervation should prevent blastema cell mitosis by abolishing expression of AEC factors, and the candidates should support regeneration to digit stages in denervated limbs.

#### 4.5.1 Candidate AEC factors

An autoradiographic study of [^3^H]‐fucose incorporation into the blastema of regenerating newt limbs found that silver grains were first detected over the basal cells of the AEC, followed by their appearance over the subjacent blastema cells, suggesting the synthesis of a glycoprotein by the AEC that diffused or was transported into the blastema interior (Chapron, 1974). Histochemical analysis for periodic acid−Schiff positive glycosylated material revealed its intracellular presence within the blastemal epidermis and extracellularly within the mesenchymal blastema (Young et al., 1985). Many growth factors are glycoproteins, several of which are expressed by the AEC and stimulate blastema cell proliferation in vitro and in vivo. Fgf1, Fgf2, Fgf8, and the anterior gradient protein (AG) are expressed by the AEC in vivo (Christensen, Weinstein, & Tassava, 2001, 2002; Han, An, & Kim, 2001; Kumar & Brockes, 2007). Blastema cells express the *bek* (FGFR2) receptor for Fgfs (Poulin & Chiu, 1995; Poulin, Patrie, Botelho, Tassava, & Chiu, 1993) and the AG receptor Prod1 (Kumar & Brockes, 2007). Fgf1 elevated the mitotic index of cultured blastema cells (Albert & Boilly, 1988; Albert, Boilly, Courty, & Barritault, 1987; Boilly, Cavanaugh, Hondermarck, Bryant, & Bradshaw, 1991), and Fgf2 elevated the mitotic index of blastema cells in amputated limbs covered by full‐thickness skin (Chew & Cameron, 1983). The only AEC candidate factors so far reported to be downregulated by denervation and to substitute for the nerve in supporting the regeneration of denervated limbs to digit stages are Fgf2 (Mullen, Bryant, Torok, Blumberg, & Gardiner, 1996) and AG (Kumar & Brockes, 2007). Fgf2 was administered in beads only to late stage blastemas. AG is involved in head development of the *Xenopus* embryo and has been the more thoroughly investigated.

The AG protein is strongly expressed in the Schwann cells insulating the axons of regenerating newt limbs at 5 and 8 days post‐amputation, when histolysis and initial dedifferentiation is under way. By 10 days post‐amputation, AG expression shifts to the gland cells of the AEC, coincident with formation of the accumulation blastema. Denervation abolishes AG expression, indicating that its expression in Schwann cells and the AEC is induced by axons. The AG gene supports regeneration to digit stages when electroporated into denervated newt limbs 5 days post‐amputation. Conditioned medium of Cos7 cells transfected with the AG gene stimulates bromodeoxyuridine (BrdU) incorporation into cultured blastema cells. This incorporation is blocked by antibodies to Prod1, suggesting that AG acts directly on blastema cells through Prod1 to stimulate their proliferation (Kumar & Brockes, 2007). Finally, AG is expressed by the AEC of regenerating aneurogenic limbs (Kumar, Delgado, Gates, Neville, Forge, & Brockes, 2011). Whether Fgf2 is expressed in the aneurogenic AEC has not been investigated.

#### 4.5.2 Nerve candidate factors

Factors expressed by DRG neurons that promote blastema cell proliferation in vitro include transferrin (Mescher & Kiffmeyer, 1992; Mescher, Connell, Hsu, Patel, & Overton, 1997), substance P (Globus & Alles, 1990; Globus, Smith, & Vethamany‐Globus, 1991), and Fgf2 (Mullen et al., 1996). Combinations of Fgf8 and bone morphogenetic protein (BMP) have also been tested as neurotrophic factors. Both are expressed in DRG neurons and are detectable in peripheral limb nerve axons in vivo (Satoh, Makanae, Nishimoto, & Mitogawa, 2016). Furthermore, they can substitute for the nerve in the outgrowth of a supernumerary axolotl limb blastema in the Lheureux model (Makanae, Mitogawa, & Satoh, 2014a).

### 4.6 The nerve and AEC express the same mitogenic factor

Glial growth factor 2 (Ggf2, neuregulin 1) (Law, Shannon‐Weickert, Hyde, Kleinman, & Harrison, 2004) is mitogenic for Schwann cells (Davies, 2000) and was suggested over 30 years ago as a nerve factor for limb regeneration (Brockes, 1984; Brockes & Kintner, 1986). It is expressed by DRG neurons, is present in the blastema, and is decreased by denervation. A newt clone of Ggf2 was briefly mentioned to rescue regeneration to digit stages in denervated axolotl limbs when injected intraperitoneally during blastema formation (Wang, Marchionni, & Tassava, 2000).

A more detailed study of neuregulin 1 (NRG1) in regenerating axolotl limbs (Farkas, Freitas, Bryant, Whited, & Monaghan, 2016) showed that transcripts of *nrg1* and its receptors *erbb2* and *erbb3* are expressed by the basal cells of the AEC and by 56% of the blastema mesenchyme cells. Antibody staining revealed expression of NRG1 and ErbB2 in dorsal root ganglia and peripheral limb nerves. Denervation of 16‐day blastemas decreased the number of *nrg1*‐expressing mesenchymal cells by 26%; the effect on *nrg1* expression by basal wound epidermis cells was not reported. Western blotting for NRG1 showed a slight drop in intensity in denervated blastemas, and the percentage of BrdU+ cells co‐localizing with NRG1 was diminished by 20%, a statistically significant reduction. Inhibition of NRG1/ErbB2 signaling by immersion of animals in mubritinib abolished blastema formation in amputated innervated limbs. Treatment of 16‐day innervated blastemas resulted in miniature regenerates, equivalent to the regenerates obtained by delaying denervation until a well‐established blastema has formed. NRG1‐soaked beads implanted under the wound epithelium of denervated limbs at 7 days post‐amputation induced blastema formation. Bead implants every 4 days from 19 to 36 days post‐amputation supported regeneration to digit stages, although not to the same degree as in innervated controls.

These results suggest a synergistic relationship between nerve, AEC, and blastema cells in which blastema cells autonomously express NRG1 in the absence of nerve, but at a level that is insufficient for mitosis. NRG1 from motor neurons would stimulate blastema cells destined to form Schwann, skeletal, and muscle cells to increase their own NRG1 expression. Sensory innervation would presumably stimulate NRG1 production in the basal cells of the wound epithelium and AEC and/or promote epidermal cell mitosis. The nerve, the AEC, and the blastema cells themselves may thus work synergistically to express a single molecule, NRG1, at a level sufficient for mitosis. This kind of synergism would easily explain why there is a 10‐fold increase in proliferation once the accumulation blastema becomes innervated. It would also explain why increasing motor innervation in the absence of sensory innervation enables complete regeneration, because the required threshold level of NRG1 could be reached in the absence of sensory nerves. The nerve addiction for regeneration that arises during limb development is thus interpreted as a quantitative increase in the requirement by blastema cells for NRG1.

Further experiments are required to assess whether (1) denervation abolishes or greatly reduces the expression of NRG1 by the AEC; (2) knocking out motor innervation maintains or increases NRG1 expression by the AEC and decreases it by blastema cells; (3) knocking out sensory innervation decreases NRG1 expression by the AEC and mitosis of epidermal cells; and (4) augmenting motor innervation in the absence of sensory innervation augments NRG1 expression by blastema cells. CRISPR knockout and knock‐in gene technology may allow greater precision in exploring the role of the nerve, AEC, and blastema cells in blastema cell mitosis.

Several other questions remain about the synergistic relationship between nerve, AEC, and blastema cells that require further research. (1) Might there be multiple redundant and synergistic circuits composed of different combinations of neural and AEC factors? (2) Can we label AEC and neural candidate factors and show that they move into the blastema and bind to receptors on blastema cells? (3) Are NRG1 and/or Fgf2 expressed in limb buds or the blastemas of amputated aneurogenic limbs? (4) If they are not, does exogenous administration of these factors render aneurogenic limbs nerve‐dependent?

Another relevant question is whether the epithelial:mesenchymal interaction that characterizes urodele limb bud development and aneurogenic limb regeneration is maintained in neurogenic limb regeneration or is completely replaced by the nerve:AEC synergy. Does the blastema mesenchyme produce a non‐neural factor necessary to maintain the AEC in addition to neural factors, as was postulated by Meinhardt (1982)? Growth‐factor‐mediated epithelial:mesenchymal interaction in urodele limb bud development and regenerating aneurogenic limbs has not been sufficiently investigated, although Fgf8 and Fgf10 are both expressed in urodele limb buds and regeneration blastemas of neurogenic limbs (Christensen, Weinstein, & Tassava, 2001; Han et al., 2001), and the expression of AG in aneurogenic limbs suggests that AG might be part of an epithelial:mesenchymal interaction in urodele limb bud development.

### 4.7 Interaction between positionally disparate cells—role of Shh and Fgf8

**Figure 4:**
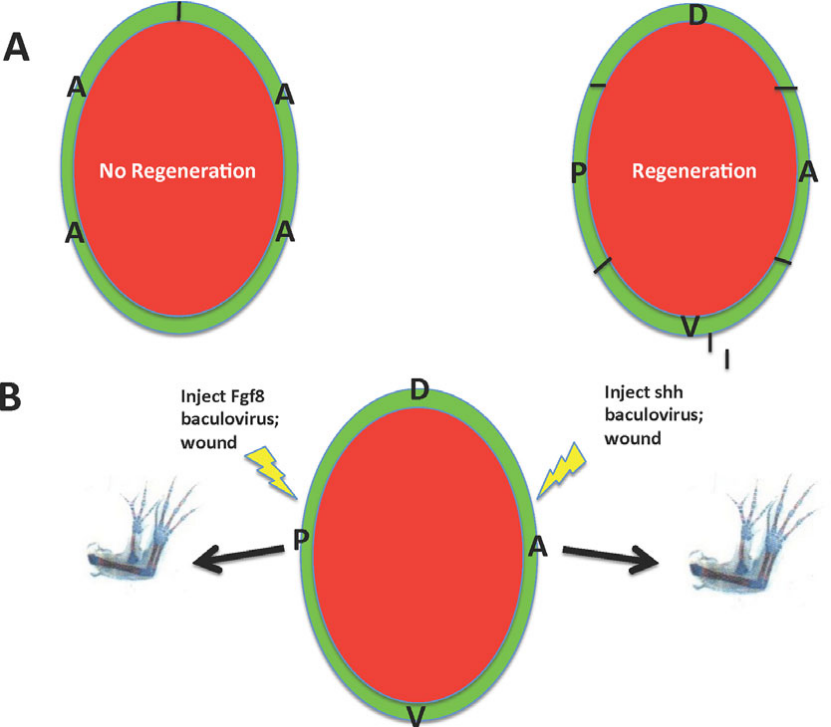
(A) Experiment showing that a longitudinal strip of unirradiated skin from one quadrant (here anterior) rotated 90^o^ and grafted as a cuff around the circumference of the amputated internal tissues of an irradiated limb (left) fails to regenerate, but if smaller unirradiated longitudinal strips from each quadrant of the limb (A, anterior; P, posterior; D, dorsal; V, ventral) are rotated and grafted (right), the limb regenerates. (B) Experiment based on Lheureux's model (1977) showing that a baculovirus construct containing the *fgf8* gene can substitute for anterior skin and a baculovirus construct containing the *shh* gene can substitute for posterior skin in evoking supernumerary limb formation at posterior and anterior wound sites on the stylopodium, respectively, to which a nerve has been deviated. S, supernumerary limb

Even in the presence of nerves and the AEC, blastema cells fail to undergo mitosis unless their transverse axial positional identities are sufficiently different to detect a discontinuity in the normal neighbor landscape. This was shown by experiments in which the normal asymmetry of newt limb skin was made symmetrical by 90° rotation of a narrow longitudinal strip of skin cut from one quadrant, grafting it around the circumference of an irradiated limb, and then amputating through the strip (Lheureux, 1975). The result was the same kind of regenerative failure seen after denervation or deprivation of wound epidermis. Normal regeneration ensued, however, after amputation through shorter longitudinal skin strips representing each quadrant that were rotated and grafted to each quadrant of the underlying tissue (Fig. 4A).

The limb bud, which in most tetrapod species develops in a posterior to anterior direction, has a posterior patch of mesenchyme called the zone of polarizing activity (Fallon & Crosby, 1975; MacCabe, Gasseling, & Saunders, 1973) that is the source of a posterior to anterior gradient of the sonic hedgehog (Shh) protein (Harfe et al., 2004; Riddle, Johnson, Laufer, & Tabin, 1993; Tickle, 2006). *Xenopus* limbs, like other tetrapod limbs, develop in posterior to anterior order (Keenan & Beck, 2016; Stopper & Wagner, 2005), and contain a posterior mesenchymal polarizing zone expressing *Shh* (Cameron & Fallon, 1977; Endo, Yokoyama, Tamura, & Ide, 1997). In these limb buds, the *shh* enhancer is hypomethylated and *shh* is expressed, but becomes hypermethylated in late tadpole limbs with loss of *shh* expression, which is correlated with regeneration of only a cartilaginous spike (Yakushiji et al., 2007). Treatment of amputated froglet limbs with an agonist of Hedgehog signaling, the synthetic small molecule Hh‐Ag, induced activation of target genes of Shh and the formation of multiple cartilaginous structures instead of a single spike (Yakushiji, Suzuki, Satoh, Ide, & Tamura, 2009). These results suggest that Shh expression is necessary for froglet limb regeneration (Yakushiji, Yokoyama, & Tamura, 2009).

By contrast, the skeletal elements of urodele limb buds and blastemas differentiate in an anterior to posterior order. Nevertheless, Shh is expressed in a patch of posterior mesenchyme (Imokawa & Yoshizato, 1997; Torok, Gardiner, Izpisua Belmonte, & Bryant, 1999), while Fgf8 is expressed in the anterior mesenchyme and the basal cells of the AEC (Han et al., 2001). Fgf8 is associated with anterior patterning of the blastema during limb regeneration in *Xenopus* (Christen & Slack, 1997). Transfection of *shh* into anterior blastema tissue of the axolotl limb by vaccinia virus results in the regeneration of supernumerary digits, but not more proximal structures (Roy, Gardiner, & Bryant, 2000).

The disparities of developmental polarity between the AP axes of urodele and other limb buds/regeneration blastemas, coupled with universal expression of *shh* in a posterior patch of polarizing mesenchyme, suggests that Shh may have a role in regeneration other than AP patterning. Shh does not appear to be necessary for stylopodial or zeugopodial AP patterning in the chick limb bud (Leitingtung, Dahn, Li, Fallon, & Chiang, 2002), but has been implicated late in limb bud development in defining digit number and identity by regulating BMP expression in the prospective chick autopodium (Dahn & Fallon, 2000; Drossopoulou et al., 2000). In regenerating axolotl limbs, inhibition of Shh signaling with cyclopamine resulted in regenerates containing all the segments distal to the amputation plane, but digital development was incomplete (Roy & Gardiner, 2000), and BMP expression was found to be independent of Shh signaling (Guimond et al., 2010). Thus Shh in limb buds and regenerating limbs appears to have a patterning role only in digital development.

Other experiments suggest a role for SHH and Fgf8 in mitosis and distalization of blastema cells. Lheureux (1977) devised an experimental system that demonstrated a synergistic effect of nerves and interaction between different APDV positional identities of skin fibroblasts in the amputated adult newt limb. He made a wound on the anterior or posterior side, or dorsal and ventral side of an adult newt stylopodium, deviated a nerve to the wound site, and juxtaposed a graft of skin from the opposite side of the limb to the skin of the wound. By themselves, nerve deviation or juxtaposing opposite positional identities resulted in the formation of blastema cells that failed to persist and divide. Together, however, they stimulated the formation of a blastema that grew and underwent morphogenesis into a supernumerary limb. Lheureux's experimental system was later used to achieve a similar result from the larval axolotl stylopodium under the name “accessory limb model” (Endo et al., 2004). These experiments also showed that the AEC forms autonomously after wounding, but requires the nerve for subsequent maintenance.

The Lheureux model was used to show that Shh can substitute for posterior skin and Fgf8 can substitute for anterior skin to evoke a supernumerary limb (Nacu, Gromberg, Oliveira, Dreschel, & Tanaka, 2016). Using a baculovirus vector system they injected *shh* under the anterior skin of the axolotl stylopodium and *Fgf8* under the posterior skin (Fig. 4B). A wound was then created in the skin and a nerve deviated to the site. The gene transfections substituted for cells of opposite positional identity. Immersion of animals with anterior skin wounds in a solution of smoothened agonist (SAG) to activate Shh signaling also produced supernumerary limbs, whereas the FGFR signaling inhibitor PD173074 blocked SAG‐induced supernumerary formation, showing that Fgf8 signaling was essential to promote Shh expression.

Since Shh is not required for patterning of skeletal elements proximal to the autopodium, these results suggest that the primary effect of Shh and Fgf8 on the AP pattern in the regenerating limb is not to act as morphogens to assign positional identity, but to promote blastema cell proliferation. Consistent with this idea, the effect of Shh on AP axial structure proximal to the autopodium in the chick and mouse limb bud seems to be primarily on cell number, since knockout experiments do not eliminate stylopodial and zeugopodial pattern (Leitingtung et al., 2002). Studies on regenerating newt limbs in which Shh signaling was inhibited by cyclopamine indicated that AP patterning is dependent on cell proliferation to expand the blastema (Singh, Doyle, Weaver, Koyano‐Nakagawa, & Garry, 2012). A major unanswered question is how neural and AEC activity is integrated with the requirement for positional disparity to stimulate mitosis and link cell proliferation to distalization, a subject that will be taken up in the section on pattern formation.

## 5 PATTERN FORMATION IN THE BLASTEMA

Pattern formation in a regenerating limb is the process of restoring a complete three‐dimensional normal neighbor map of positional identities from the progeny of cells derived from the level of amputation (Mittenthal, 1981). Cells differentiate in accordance with their new positional specifications to restore the original structure of the limb. The mechanisms of pattern formation in regeneration are still largely a mystery, but we have gained insights via studies of gene expression associated with the different stages of regeneration, and grafting experiments in which the spatial relationships of limb and blastema tissues are altered.

### 5.1 Genes associated with pattern specification

The axial patterns of regenerating tissues are set up during blastema formation and growth, and are associated with the expression of a number of genes that are also expressed during limb bud development. Several homeobox genes encoding transcription factors are expressed in growing blastemas derived from the proximal stylopodium (Gardiner & Bryant, 1996; Gardiner, Blumberg, Komine, & Bryant, 1995; Geraudie & Ferretti, 1998). Forelimb identity is associated with the *tbx5* gene and hindlimb identity with the *tbx4* gene (Simon et al., 1997). The expression pattern of *Hoxa* gene combinations (*Hoxa* codes) is considered to indicate the order of specification of the new positional identities representing the PD axis of the limb. *Hoxa‐9*, *Hoxa11*, and *Hoxa‐13* are expressed serially in proximal to distal temporal and spatial order in the mesenchyme of the blastema (Roensch, Tazaki, Chara, & Tanaka, 2013). *Hoxa9* is expressed first throughout the blastema, *Hoxa11* next in the prospective zeugopodial region, and *Hoxa 13* last in the prospective autopodial region. *Meis 1* and *2* are two other genes expressed preferentially in the prospective stylopodial region. These genes are upregulated in autopodial blastemas proximalized by RA, while *Hoxa13* is downregulated, and autopodial blastemas are also proximalized by overexpression of *Meis 2* (Mercader, Tanaka, & Torres, 2005). These observations implicate *Hoxa9/Meis 1/2* in specfying the PD pattern of the stylopodium, *Hoxa9/11/Meis2* the zeugopodium and *Hoxa9/13* the autopodium. *Hoxd‐8*, ‐*10* and ‐*11* are associated with AP patterning in limb buds, and are also expressed in the regeneration blastema (Torok, Gardiner, Shubin, & Bryant, 1998). *Hoxd‐10* expression is upregulated by RA, suggesting that it might play a role in maintaining posterior positional identity.

### 5.2 Transverse axial reversal experiments and models of pattern formation

#### 5.2.1 AP or DV reversal

Reversal of either the AP or DV axis evokes a maximum of two supernumerary limbs where graft tissue confronts host tissue on the anterior and posterior (AP reversal) sides of the limb, or the dorsal and ventral sides (DV reversal). Supernumeraries form most frequently after AP reversal and are mirror imaged to the primary regenerate developed from the graft (i.e., they have host limb handedness). Supernumeraries form less frequently after DV reversal of early or medium bud blastemas or limb bud tips, but at high frequency after DV reversal of palette stage blastemas (Bryant & Iten, 1976; Iten & Bryant, 1975; Maden, 1980a; Maden & Turner, 1978; Tank, 1978; Thoms & Fallon, 1980; Wallace & Watson 1979).

Grafts between differently marked (ploidy, pigmentation) blastemas and limb stumps have shown that host and graft can make equal or variable cellular contributions to the supernumerary (Muneoka & Bryant, 1984; Stocum, 1982; Thoms & Fallon, 1980). There is probably some mixing across the boundary of the two contributions, since regenerates derived from surgically constructed asymmetric limbs that are one half triploid revealed triploid cell migration for a short distance across the midline (Tank, Connelly, and Bookstein, 1985).

#### 5.2.2 APDV reversal

The results of reversing the AP and DV axes simultaneously by 180^o^ inversion of the blastema on its limb stump are more complex. Supernumeraries can be evoked by as little as 20^o^ rotation, and the frequency of cases forming them increases with the angle of rotation up to a maximum at 180^o^. Up to three supernumeraries can be formed simultaneously whose loci and handedness are variable (Maden & Turner, 1978; Tank, 1981; Turner, 1981; Wallace, 1978; Wallace & Watson, 1979).

Supernumerary limbs of high complexity also arise after 180^o^ rotation of skin and muscles, or cross‐transplant of muscles and amputation through the grafted region (Carlson, 1974, 1975a, b), as well as after wounding unamputated limbs coupled with nerve deviation, or following implants of carcinogens or non‐limb tissues. Presumably the mechanisms underlying the development of these supernumeraries involve the same kinds of interactions between fibroblasts of differing positional identities as those operating after blastema axial reversals.

#### 5.2.3 Models of pattern formation

**Figure 5:**
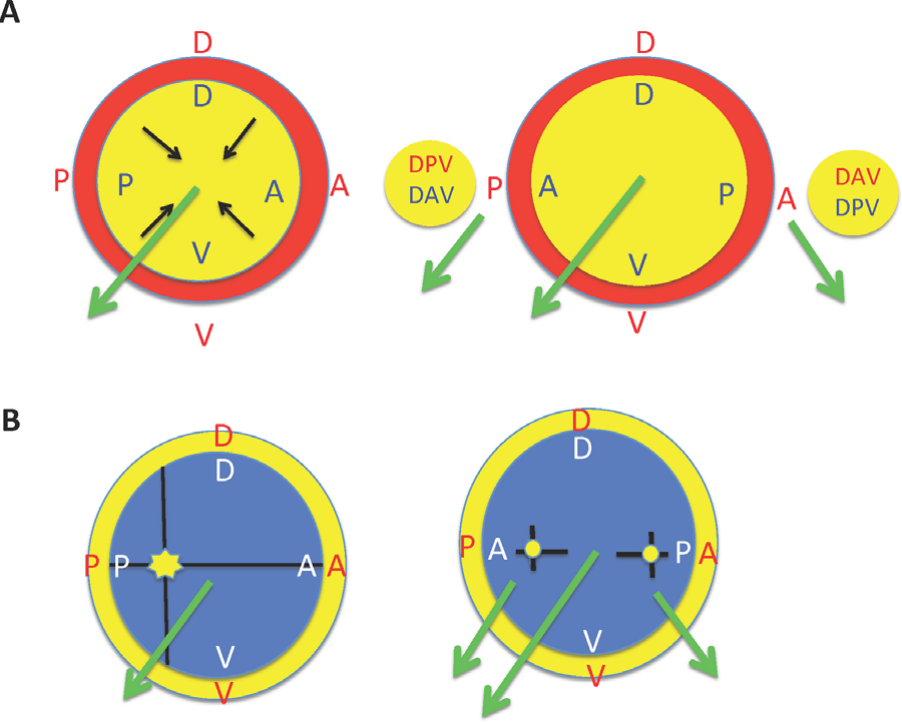
Supernumerary formation. (A) Polar coordinate model. Red, stump; yellow, blastema. Left, normal regeneration. Circumferential fibroblasts interact centripetally (black arrows) to initiate regenerative outgrowth (green arrow). Right, reversal of the blastema AP axis allows interactions between anterior and posterior halves of stump and graft tissues (DPV/DAV, DAV/DPV) to regenerate two supernumerary limbs with stump handedness (shorter green arrows); the graft develops (longer green arrow) with the handedness of origin. (B) Boundary model. Left, normal regeneration. Yellow circle, stump; blue circle, blastema. Interaction between cells at a posteriorly located intersection between AP and DV boundaries triggers the production of a morphogen (star) that initiates regeneration. Right, Reversal of the blastema AP axis creates supernumerary loci of morphogen production (yellow circles) on the anterior and posterior sides of the limb. The primary limb (longer green arrow) has graft handedness and the two supernumerary limbs (shorter green arrows) have stump handedness

Two prominent models of blastema patterning have been proposed based on how well they predict the number, location, and handedness of these supernumeraries. These are the polar coordinate model (Bryant, French, & Bryant, 1981; French, Bryant, & Bryant, 1976) (Fig. 5A) and the boundary model (Meinhardt, 1983a) (Fig. 5B).

The polar coordinate model is an abstract model that assigns dermal fibroblasts angular and radial coordinates representing their position. The angular value represents their position on the circumference (often illustrated as a series of “clock face” numbers) and the radial value represents their position on the PD axis. Following amputation and histolysis, blastema cells having the value of their PD level of origin migrate centripetally from different positions on the limb circumference (Gardiner, Muneoka, & Bryant, 1986) and use short range interactions to intercalate a complete cross‐section of APDV identities which adopt the next distal PD value. Beryllium treatment of amputated limbs induces pattern abnormalities by interfering with the migration and interaction of blastema cells derived from different circumferential positions on the skin (Cook & Seifert, 2016). Successive rounds of migration and intercalation after amputation restore the normal APDV and PD nearest neighbor map. Confrontation of cells at AP or DV junctions after axial reversal evokes short arc intercalation of a supernumerary set of circumferential values that drive the formation of an accessory blastema (Bryant et al., 1981; French et al., 1976). The model correctly predicts the number, location, and handedness of the supernumerary limbs formed after AP or DV reversal of the blastema.

The boundary model (Meinhardt, 1983a) postulates four structural domains differing in positional identity within the cross‐section of the amputation surface. Large anterodorsal and anteroventral domains confront smaller posterodorsal and posteroventral domains. The DV boundary evenly divides dorsal and ventral, whereas the AP boundary is located more posteriorly. Where these boundaries intersect, interactions take place that induce the expression of a diffusable morphogen(s). It might be presumed that this is Shh and Fgf8 but, as argued earlier, these signals more probably mediate mitosis and distalization, with a direct patterning effect of Shh only on the autopodium. Possible mechanisms for generation of the APDV pattern and distalization are discussed later.

The boundary model makes exactly the same predictions as the polar coordinate model with regard to supernumeraries generated by AP or DV axial reversal, because reversal of either the AP or DV axis creates new sets of intersecting AP and DV boundaries. Following reversal of the blastema AP axis, two zones of intersecting boundaries are established on anterior and posterior sides of the limb in addition to the original (primary) set; supernumeraries can now arise from both locations via mitosis and distalization.

After APDV axial reversal, the polar coordinate model predicts that two anatomically normal supernumeraries will arise, one posterodorsally and one anteroventrally; one of these will have stump handedness and the other graft handedness (Bryant & Iten, 1976). These locations are dictated by the fact that they are the only ones at which APDV positional identities can intercalate a complete transverse pattern. However, three other structural classes are also produced: mirror imaged (double dorsal or double ventral), part normal/part mirror imaged, and part normal/part inverted (mixed‐handed) limbs (Maden, 1980a; Maden & Mustafa, 1982a; Papageorgiou & Holder, 1983). These classes are not predictable by the polar coordinate model, but the boundary model could correctly predict most of them (Maden, 1983).

### 5.3 Regeneration of half and double half limbs

**Figure 6:**
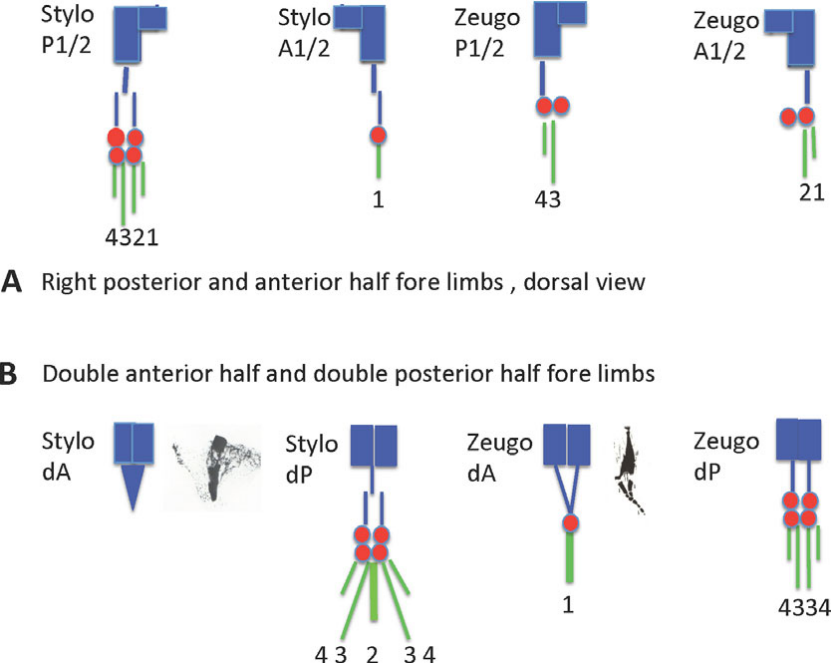
Regeneration of anterior and posterior half and double half stylopodia and zeugopodia of axolotl forelimbs. Stylopodium and zeugopodium, blue; carpals, red; digits, green. (A) Half limbs. Posterior and anterior stylopodial halves exhibit non‐equivalent regeneration, with posterior halves regenerating much more than anterior halves, whereas regeneration of posterior and anterior half zeugopodia is more equivalent. (B) Double half limbs. A similar non‐equivalence is exhibited by double anterior stylopodia and zeugopodia

The results of amputating half (Fig. 6A) and double half (Fig. 6B) limb constructs have revealed non‐equivalencies in regenerative potential between limb halves that give insights into their relative contributions to the blastema.and their interactions during regeneration.

#### 5.3.1 Half limbs

The creation of half stylopodia by irradiation or surgical deletion has shown that the posterior half of the stylopodium has the potential to regenerate a whole limb, whereas the anterior half is able to regenerate an anterior half zeugopodium. The posterior half most often regenerates all of the digits, whereas only the anterior‐most digit is regenerated by the anterior half (Stocum, 1978; Wigmore & Holder, 1985, 1986). Dorsal and ventral half stylopodia regenerate with the normal AP pattern and number of digits, but are deficient in ventral and dorsal muscle, respectively (Maden, 1979a, b; Wigmore & Holder, 1986). Posterior half and anterior half zeugopodia of forelimbs each regenerate their half of the zeugopodium plus half the digits (Goss, 1957a, b; Stinson, 1963, 1964a, b, c). Dorsal and ventral half forelimb zeugopodia regenerate limbs normal in the AP axis, but deficient in ventral and dorsal muscle, respectively (Wigmore, 1986).

#### 5.3.2 Double half limbs

Double anterior or posterior half zeugopodia are made by splitting the left and right limb between the two zeugopodial skeletal elements and exchanging the posterior and anterior halves. Double anterior, posterior, dorsal and ventral stylopodia, as well as double dorsal and ventral zeugopodia, are made by grafting together the skin and muscle from the same halves of right and left limbs, because splitting skeletal elements in the AP and DV planes of the stylopodium and DV plane of the zeugopodium is not practical. The presence of asymmetric skeletal elements in the construct does not matter, however, since the skeletal elements are derived from dermal fibroblasts.

Amputated double anterior stylopodia regenerate only a symmetrical tapered cone of cartilage, whereas double posterior stylopodia regenerate double posterior limbs with a symmetrical distal stylopodium, two ulnae or fibulae and six (forelimb) to eight (hindlimb) digits, with some fusion of structure in the midline (Bryant, 1976; Bryant & Baca, 1978; Holder, Tank, & Bryant, 1980; Krasner & Bryant, 1980; Stocum, 1978; Tank, 1979). Double dorsal and double ventral stylopodia regenerate double dorsal and double ventral limbs, respectively, including symmetrical muscle patterns (Burton, Holder, & Jesani, 1986; Ludolph, Cameron, & Stocum, 1990). Double anterior zeugopodia regenerate two radii or tibias that converge distally, one to two carpals or tarsals and a single symmetrical digit, in contrast to double posterior zeugopodia which regenerate a double ulna or fibula and two symmetrical sets of posterior digits (Bryant & Baca, 1978; Holder et al., 1980; Krasner & Bryant, 1980; Stocum, 1978).

The polar coordinate model explains the differences in regeneration of digit number between anterior versus posterior half stylopodia and between double anterior and double posterior stylopodia by assigning more circumferential positional identities to the posterior half of the limb. As a result of fewer identities, successive rounds of circumferential interactions in anterior half or double anterior stylopodia quickly converge to a uniform anterior identity, halting distalization at a stylopodial level (Bryant et al., 1981). This would be akin to the Lheureux experiment providing irradiated limbs with only anterior skin in their circumference so that any positional difference is insufficient to maintain regeneration. Since fewer PD structures have to be regenerated in double anterior zeugopodia, a single symmetrical digit can be regenerated under these circumstances.

The boundary model explains the regeneration of only a single digit by the anterior half and the pattern convergence of double anterior stylopodia and zeugopodia by the lack of a posterior AP boundary. This boundary would be present in posterior half and double posterior limbs. The fact that there is regeneration of anterior half stylopodia and zeugopodia, and double half anterior zeugopodia, might be due to an autonomous regenerative capacity of anterior limb tissue. Evidence for this possibility is that exogenous Fgf8 can evoke single digit formation from anterior limb tissue in the Lheureux model (Nacu et al., 2016). The regeneration of dorsal and ventral half limbs and double dorsal and ventral limbs is explained by the boundary model as well, since these constructs would contain the posteriorly located AP boundary. There is no convergence of positional identity in the DV axis, or, if there is, it has no effect on distalization, suggesting that a DV boundary either does not exist or is unimportant to either AP or DV patterning. DV patterning might also be achieved by a mechanism different from that of AP patterning; the results of experiments with the *Xenopus* limb bud indicate that asymmetric expression of DV homeobox genes, particularly *Lmx*, is instrumental in patterning the DV axis of the blastema (Matsuda, Yokoyama, Endo, Tamura, & Ide, 2001; Shimokawa, Yasutaka, Kominami, & Shinohara, 2013).

Interestingly, the healing time allowed between creating double half stylopodia and amputation is a major factor in their ability to regenerate. Double anterior stylopodia fail to regenerate regardless of the length of time elapsed between making the construct and amputation. Double posterior stylopodia, however, lose regenerative capacity in proportion to healing time. Double posterior hindlimb stylopodia of small (60−80 mm) axolotl and *Ambystoma tigrinum* larvae healed for 10−14 days regenerated an average of 2.13 fibulae, 9.25 tarsals, and 6.63 toes, whereas after a 32‐day healing period they regenerated an average of 1.33 fibulae, 5.33 tarsals, and 3.00 toes (Stocum, 1978). There was no effect of healing time on either double anterior or double posterior zeugopodia. Krasner and Bryant (1980) also did not detect any effect of healing time on double posterior zeugopodia of adult newt limbs. In a more detailed study of the effect of healing time on somewhat larger axolotl larvae, regenerative capacity of double posterior stylopodia decreased rapidly to near zero from 5 to 30 days of healing time (Tank & Holder, 1978), whereas amputating the constructs at the same time they were made resulted in the regeneration of symmetrical double posterior limbs (Holder et al., 1980). Secondary and tertiary amputations of these regenerates resulted in expanded digit numbers. So far, there has been no satisfactory explanation as to why healing time or age has these effects.

### 5.4 Skin fibroblasts play the major role in regenerate patterning

Goss (1957b) showed that anterior or posterior half zeugopodia regenerated half limbs, but regenerated complete limbs after removal of one half of the internal limb tissues while retaining a full circumference of skin. Wigmore and Holder (1986) replaced half the limb skin of the stylopodium or zeugopodium with head skin, which does not support limb regeneration. Stylopodia with their posterior half skin replaced with head skin behaved like anterior half limbs, regenerating a high proportion of single anterior skeletal elements. Replacement of anterior, dorsal, and ventral half skin of stylopodia with head skin resulted in only minor defects in skeletal pattern, but there were ventral muscle deficiencies after replacement of dorsal and ventral half skin. No defects were observed after replacement of any zeugopodial half circumference of limb skin with head skin. Maden and Mustafa (1982b) performed a series of experiments in which the skin was made symmetrical with respect to the internal tissues of the limb. The frequency of symmetrical regenerates in stylopodial and zeugopodial constructs with symmetrical skin on normal internal tissues was highest with double posterior skin (49%), followed by double dorsal skin (28%), double anterior skin (18%), and least (7%) with double ventral skin. These results suggest that posterior and dorsal skin fibroblasts are responsible for most of the regeneration potential of the limb (Bryant, Gardiner, & Muneoka, 1987; Holder, 1989). Theoretically, since the regenerate cartilage is derived by transdifferentiation of dermal fibroblasts, the cartilage in these double dorsal and ventral regenerates should be symmetrical as well.

### 5.5 Retinoid‐treated normal, half, and double half limbs

#### 5.5.1 Retinoid treatment of normal amputated limbs

**Figure 7:**
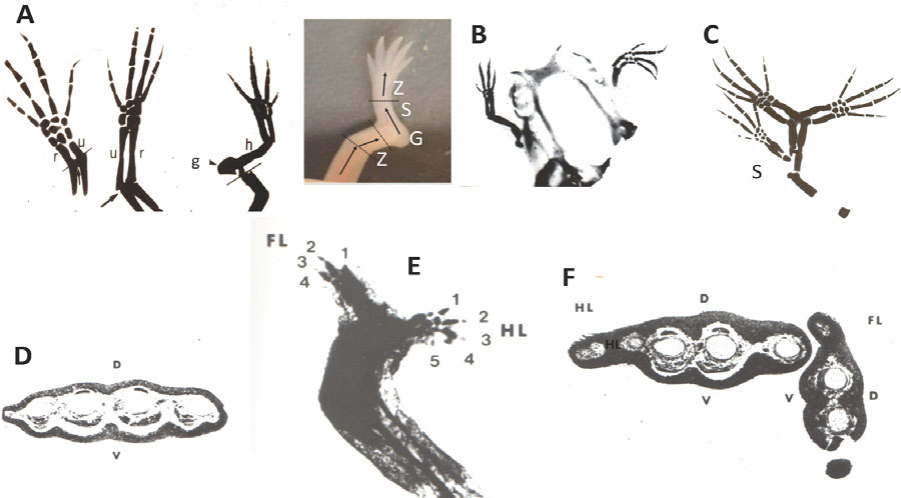
RA‐treated amputated normal limbs, anterior half, dorsal half, double anterior half and double dorsal half limbs with retinoic acid. (A) Normal limb. Left to right, increasing dose of RA after amputation through the distal radius/ulna. r = radius, u = ulna, h = humerus; g = girdle. line = level of amputation. Color photo shows RA‐induced serial PD duplication of hind limb segments after amputation through the distal zeugopodium (Z). G, S, Z indicate the duplicated girdle, stylopodium, and zeugopodium/autopodium. (B) Regeneration from RA‐treated anterior half zeugopodia grafted to the orbit. AP/DV complete, proximalized limbs were regenerated. (C) Regeneration of mirror‐image limb from RA‐treated double anterior zeugopodia. A proximalized supernumerary limb (S) arose where tissue of the grafted anterior half met posterior tissue. (D) Section through a regenerated RA‐treated dorsal half zeugopodium at the level of the distal metacarpals. RA proximalizes these regenerates and ventralizes positional identity, as shown by the normal pattern of extensor muscles on the dorsal side (d) and flexor muscles on the ventral side (v). (E) RA‐treated double dorsal half zeugopodium. One half was fore limb, the other half was hind limb. A fore limb (FL) was regenerated by the forelimb half and a hind limb (HL) by the hindlimb half. Numbers indicate digits. (F) Cross‐section through another such specimen showing that both the forelimb and hind limb regenerated normal DV muscle patterns. After Thoms and Stocum (1984), Kim and Stocum (1986a), and Ludolph et al. (1990)

Retinoids are able to reprogram the positional identity of blastema cells, as first discovered in the 1970s by Niazi and colleagues in regenerating toad tadpole limbs (see Niazi, 1996, for a review). Retinol palmitate mixed into the water caused the formation of multiple regenerates proximalized in the PD axis. Two other methods of retinoid delivery have also been used: implanting a matrix containing the retinoid at the base of the developing blastema (Keeble & Maden, 1989) and intraperitoneal injection of the retinoid into the body cavity (Thoms & Stocum, 1984). In urodeles, retinoids do not cause multiple limb formation, but when administered during blastema formation do proximalize the blastema cells so that a blastema derived from the distal zeugopodium will serially duplicate more proximal structures from that level in a dose‐dependent manner (Maden, 1982a; Niazi, Pescetelli, & Stocum, 1985; Thoms & Stocum, 1984 **)** (Fig. 7A). The effect is local to cells at the amputation level. Distal migration of cells from proximal levels of the limb was ruled out by the fact that serial duplication of proximal structures is observed in regenerates derived from RA‐treated PD reversed limbs (Wallace & Maden, 1984). Retinoids work by entering cells and binding to a cellular retinoic acid binding protein (CRABP) (Keeble & Maden, 1989; McCormick, Shubeita, & Stocum, 1988), which translocates them to the nucleus where they bind to and activate retinoic acid receptors (RARs). These receptors belong to the steroid hormone/thyroid hormone nuclear binding superfamily. In turn, the RARs bind to retinoic acid response elements in the regulatory regions of target genes to alter transcriptional activity (Allenby et al., 1993; Di Masi et al., 2015).

Using retinoid‐impregnated silastin blocks implanted under the skin at the base of the developing blastema, Keeble and Maden (1989) surveyed the effectiveness of several natural and synthetic (derived from RA) retinoids to proximalize blastema cells. Their general finding was that alterations of the polar end group of RA to produce esters or the alcohol or aldehyde forms of RA abolish the ability to proximalize positional identity, whereas alterations of the ring or side chain to produce the derivatives TTNPB and arotinoid greatly enhance this ability. TTNPB was 100× more effective than RA at producing serial PD duplications (Keeble & Maden, 1989) and arotinoid delivered intraperitoneally was 50× more effective (Kim & Stocum, 1986c). These retinoids are also more toxic, however, and thus RA has become the tool of choice to reprogram positional identity. There was no consistent effect of RA, TTNPB, or arotinoid on AP or DV pattern except for occasional extra spikes of cartilage although, in one case of a regenerating hindlimb treated with arotinoid by intraperitoneal injection, two PD‐duplicated limbs mirror imaged in the DV axis were regenerated (Kim & Stocum, 1986c). Administration of RA at later stages of regeneration results in abnormalities or inhibition of regeneration (Niazi, 1996; Niazi, Pescetelli, & Stocum, 1985). Interestingly, although paedomorphic axolotls are induced to metamorphose by injecting them with thyroxine, animals co‐injected with thyroxine and RA do not metamorphose and their amputated zeugopodia are more strongly proximalized than by an equivalent dose of RA, suggesting that RA and thyroxine exert their effects through similar and perhaps competitive pathways (Crawford & Vincente, 1998).

The fact that RA can proximalize positional identities of blastema cells argues that it might be an important component of the molecular mechanism that patterns the blastema. This idea is supported by several lines of evidence. First, RA is present in posterior–anterior and distal to proximal gradients within the blastema, which is what might be expected given that RA proximalizes distal blastema cells (Scadding & Maden, 1994) and simultaneously posteriorizes them (see later). Second, CRABP levels are significantly higher in the blastemas of RA‐treated limbs (Keeble & Maden, 1986). Third, the inhibitor of RA synthesis, disulfiram, has detrimental effects on formation of the wound epidermis and inhibits limb regeneration when administered at very early stages of limb regeneration (Lee, Ju, & Kim, 2012). Fourth, RA‐induced proximalization activates a transgenic RA reporter gene in the fibroblast contribution to the blastema (Monaghan & Maden, 2012), exactly what is predicted from the evidence that the positional identities of fibroblasts are the basis for the rule of distal transformation (Nacu et al., 2013). Fifth, five RA receptor isoforms (RARs) have been detected in the blastema: α1, 2, δ1a, b and δ2 (Maden, 1997, 1998). The functions of three of these isoforms have been determined by constructing chimeric RARs with ligand binding domains substituted with the ligand binding domain of a thyroid hormone (TH) receptor. The chimeric receptors and a retinoic acid response element–reporter gene construct were then cotransfected into cultured blastema cells, activating target genes. In this way, it was shown that the α1 receptor mediates RA‐induced growth inhibition, the δ1 receptor mediates changes in the secretory properties of the epidermis (Ragsdale, Hill, Gates, & Brockes, 1992; Hill, Ragsdale, & Brockes, 1993; Schilthuis, Gann, & Brockes, 1993) and the δ2 receptor is responsible for proximalization of positional identity (Pecorino, Entwhistle, & Brockes, 1996). Sixth, inhibition of the δ2 receptor at medium bud−late bud stages of axolotl limb regeneration aborts regeneration, whereas other RAR antagonists have no effect (Del‐Rincon & Scadding, 2002). Seventh, transcriptomic analysis indicates that RA upregulates proximal Hoxa and Meis gene expression as well as δ1 receptor transcripts and silences distal genes in regenerating axolotl limbs, whereas treatment of early blastemas with a selective agonist that activates the δ2 receptor results in proximalization and serial duplication of limb structure (Nguyen et al., 2017).

The effects of RA on positional identity in the AP and DV axes were revealed when zeugopodial halves were amputated and treated with RA at the same dose that causes maximum proximalization of pattern in normal limbs. Distally amputated control anterior and dorsal half zeugopodia regenerated as half limbs. By contrast, the blastemas of RA‐treated anterior and dorsal half zeugopodia completed the complementary posterior and ventral half patterns, respectively, while simultaneously duplicating stump structures in the PD axis (Fig. 7B, C) (Kim & Stocum, 1986a; Ludolph et al., 1990; Stocum & Thoms, 1984). The regenerates of RA‐treated anterior or ventral half zeugopodia are thus identical to those of amputated normal zeugopodia treated with RA.

Even more strikingly, distally amputated double anterior and double dorsal zeugopodia treated with RA at a dose that proximalizes the normal limb to the level of the girdle produced two mirror‐imaged regenerates with normal transverse pattern and duplicated in the PD axis (Fig. 7D), whereas RA‐treated posterior and ventral half limbs and double posterior and double ventral limbs failed to regenerate (Kim & Stocum, 1986a; Ludolph et al., 1990). These results showed that RA not only proximalizes positional identity of blastema cells in amputated normal limbs, it simultaneously posteriorizes and ventralizes positional identity. Furthermore, ventralization by RA has been shown in the Lheureux model by the formation of a supernumerary limb after grafting RA‐treated dorsal skin to a dorsal wound with a deviated nerve (Satoh & Makanae, 2014). In half or double half limbs treated with RA, the results are explained by posteriorized and ventralized blastema cells coming into contact with adjacent anterior or dorsal stump cells that are unaffected by RA, leading to the formation of what are essentially two “supernumerary” limbs, as would be predicted by both the polar coordinate and the boundary model. The fact that amputated normal limbs proximalized to the level of the girdle arise from the anterodorsal quadrant of the distal zeugopodial stump is consistent with this conclusion, because this is the only location at which cells with A, P, D, and V positional identities and an AP boundary are available to interact. Since RA simultaneously posteriorizes and ventralizes positional identity, we may speculate that these effects are also mediated by the δ1 receptor.

RA‐induced proximalization and posteriorization of positional identity after amputation through the distal end of double anterior zeugopodia is dose‐dependent, as indicated by the increasing degree of AP/PD duplication with higher RA dose per gram of body weight (Monkmeyer, Ludolph, Cameron, & Stocum, 1992). Control double anterior regenerates formed an average of two digits. The lowest dose of RA used (20 μg) resulted in two mirror‐image autopodia with a total average digit number of 4.5 while duplicating only the distal end of the zeugopodium, whereas 100−150 μg evoked two mirror‐image limbs with seven to eight digits and serially duplicated to the girdle, with gradual increases at intermediate doses. The regeneration of double posterior limbs treated with progressively higher doses of RA was inhibited. Presumably, similar results would be obtained with double dorsal and ventral limbs, although this has not been verified.

The histological features of blastema development in RA‐treated normal and double anterior and double posterior limbs are of interest (Ju & Kim, 1994; Kim & Stocum, 1986b; Stocum & Crawford, 1987). In normal limbs, an initial blastema cell accumulation is formed but then disappears, followed by an extended period of histolysis proximally compared to untreated controls. Expression of the lysosomal protease cathepsin D and of trypsin and chymotrypsin‐like activity is enhanced (Ju & Kim, 1998; Lee & Kim, 1996), but MMP‐9 is downregulated (Yang et al., 1999). The effect of RA on other proteases has not been investigated. Fgf8 is expressed in distal limb tissues over a time period that coincides with the period of histolysis (Han & Kim, 2002).

**Figure 8:**
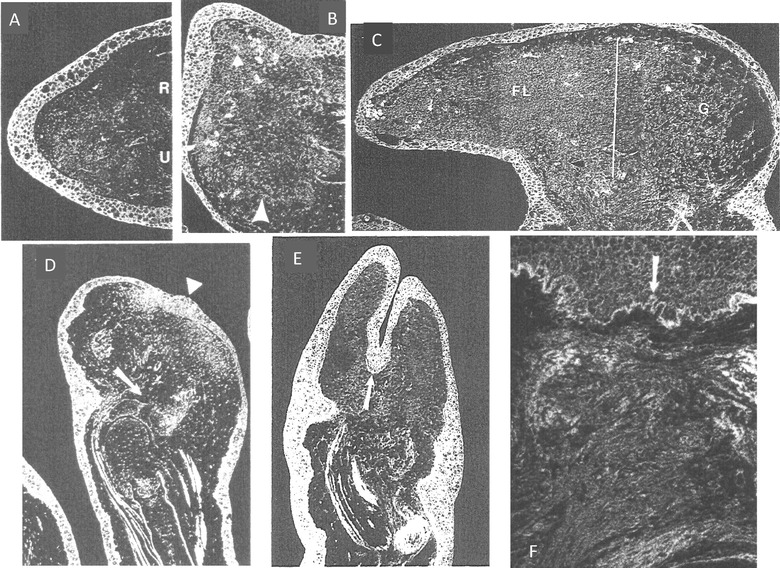
Histology of RA‐treated limbs. (A) Normal fore limb blastema, no RA treatment. R = radius; U = ulna. (B) RA‐treated medium bud stage blastema, growing posteriorly. Arrowhead indicates proximal high density region of blastema cells. (C) RA‐treated late bud blastema growing perpendicular to the PD axis of the stump. White line, transition between prospective girdle (G) and the free limb (FL). (D) RA‐treated double anterior zeugopodium showing twin condensations of blastema cells separated by thickened epidermis (arrowhead) Arrow points to remnant of zeugopodial stump cartilages. (E) Proximalized twin blastemas growing from a RA‐treated double anterior limb. Arrow indicates basement membrane reforming beneath the epidermis between the blastemas, but not under the rest of the blastemal epidermis. (F) Non‐regenerating RA‐treated double posterior limb with thick basement membrane (arrow) and connective tissue pad under the epidermis. After Kim and Stocum (1986b)

Subsequently, a blastema emerges that consists of a low‐density population of blastema cells adjacent to the zeugopodial cartilages and a distal high‐density population that forms under an AEC pointing posteriorly to the PD axis (Fig. 8A). The low‐density cell population bulges out on the anterior side of the limb and gives rise to the girdle. The high‐density population gives rise to the free limb, which angles posteriorly across the longitudinal axis of the limb as it grows (Kim & Stocum, 1986b). The blastema assumes the shape, proportions, and growth characteristics of a blastema derived from the stylopodium (Holder & Reynolds, 1984). Similar histological changes have been confirmed and changes to the skin revealed by additional electron microscope observations made of regenerating limbs of axolotls treated by immersion in solutions of retinol palmitate (Scadding, 1990).

The increased density of the distal part of the blastema in RA‐treated limbs compared to controls is suggestive of changes in the ECM related to blastema cell adhesivity. As indicated earlier, RA administered to axolotls in an affinophoresis assay abolishes the sorting behavior of wrist and elbow forelimb blastemas grafted to the blastema‐stump junction of a hindlimb regenerating from the mid‐femur level, and intercalary regeneration is abolished after grafting an RA‐treated wrist blastema to a mid‐stylopodial hindlimb stump (Crawford & Stocum, 1988b). Very little work has been done to explore such changes, except for the finding of Maden and Keeble (1987) of increased levels of fibronectin in the blastemas of retinoid‐treated limbs.

After amputation through a double anterior distal zeugopodium, double blastemas consisting of proximal low‐density and distal high‐density blastema cells formed over each of the zeugopodial cartilage elements after a prolonged period of dedifferentiation, each with its own AEC (Fig. 8B). These blastemas grew with stylopodial blastema characteristics but, in contrast to RA‐treated normal limbs, they grew straight out of the limb stump and formed limbs mirror imaged in the AP axis. Interestingly, between the twin blastemas a small area of basement membrane was reconstituted, but not under the blastema AEC, suggesting differences in MMP expression between these two regions. RA‐treated double posterior limbs did not form a blastema, similar to the results of grafting full‐thickness skin over limb stumps (Chew & Cameron, 1983; Mescher, 1976; Tassava & Garling, 1979) or providing only skin from one quadrant of the limb to provide blastema cells (Lheureux, 1975). A basement membrane and thick mat of connective tissue was quickly formed under the wound epidermis (Kim & Stocum, 1986b) (Fig. 8C). This result suggests that positional disparity is a distinct requirement for MMP production by the wound epidermis and macrophages to prevent re‐formation of the basement membrane, but the cellular and molecular mechanism by which this works is unknown.

In the three or more decades since experiments on half and double half limbs were carried out, no further work on them has been done. The molecular biology of limb regeneration has advanced rapidly, however, and it would be instructive to repeat these experiments assessing the expression of ECM molecules, proteases, signaling molecules, and blastema cell molecular markers. For example, what does the expression pattern of proteases *Shh*, *Fgf8* and *Hoxa* and *d* genes look like in the blastemas of regenerating double half limbs treated with RA?

### 5.6 Mechanisms of distalization

#### 5.6.1 Distalization after simple amputation

**Figure 9:**
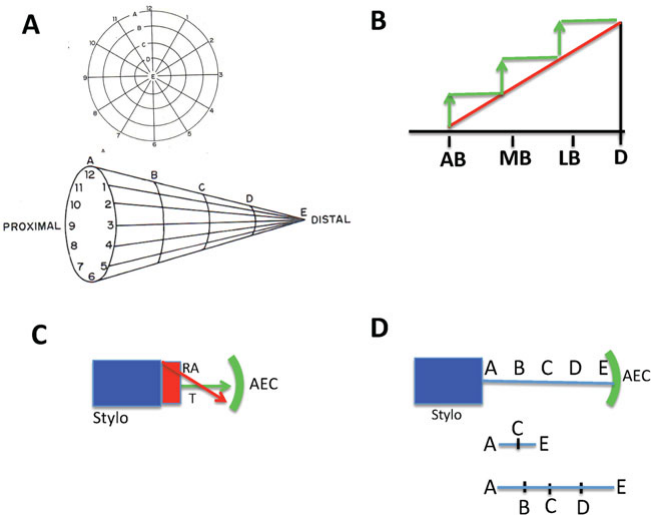
Models of distalization. (A) Polar coordinate model. Top, planar representation. The concentric circles (radial values) labeled A−E represent the PD positional identities generated by successive reiterations of centripetal migration and interaction. Numbers represent angular values. Bottom, the radial values telescoped out as each of the radial values is realized. (B) “Bootstrap” model. Red line, morphogen levels from proximal to distal. Green line, production of an AEC factor that increases morphogen levels in a proximal to distal direction. AB, accumulation blastema; MB, medium bud; LB, late bud; D, digits. (C) Regeneration of the segment of amputation (distal stylopodium, red), driven by a high level of RA (red arrow) that drops off distally to interact with a mitotic timing mechanism to specify remaining PD positional identities. (D) Intercalary averaging mechanism. Missing positional identities represented as A−E. The first step is intercalation of the intermediate positional identity; successive intercalations complete the PD sequence. (A) After French et al. (1976) and Bryant et al. (1981). (D) After Maden (1977)

There are several models of distalization after simple amputation through the upper stylopodium (Fig. 9). The positions of blastema cells in the polar coordinate model are given by angular and radial coordinates in which the angular coordinate identifies position on the limb circumference and the radial coordinate position on the PD axis (Bryant et al., 1981; French et al., 1976). Distalization requires the interaction of blastema cells with differing angular coordinates migrating centripetally from the circumference across the amputation surface to intercalate a complete cross‐section of angular identities that adopt the next positional value in the PD sequence (Fig. 9A). Repetition of this process restores the complete normal neighbor map. After amputation through the stylopodium, each repetition would specify stylopodial PD values until the elbow is reached, whereupon there would be a split into two circles of identities for the radius and ulna that would continue the process until further splits took place into circles that represent autopodial elements. It is important to understand that the radial values in the planar depiction of the model (upper part of Fig. 9A) represent the successive PD positional identities that are regenerated by these interactions, not a literal map of the identities on the amputation surface. The interactions between migrating cells after amputation would couple mitosis with distalization.

Meinhardt (1983b) proposed a “bootstrap” model to restore the PD axis in which distalization is driven by the production of an AEC morphogen (Fig. 9B). Higher concentrations of the morphogen specify more distal positional identities. The blastema cells produce an AEC maintenance factor (AECMF) that controls production of the AEC morphogen. The concentration of morphogen is lowest at the earliest stages of regeneration and will specify the most proximal structure to be regenerated. Once specified, these cells now ramp up their production of AECMF, resulting in production of a higher concentration of morphogen by the AEC that specifies the next more distal structure, and so on. Progressively more distal PD positional identities are thus serially “bootstrapped” into existence by stepwise increases in AECMF and AEC morphogen. Combined with the polar coordinate model, bootstrapping gives a physical mechanism for assignation of progressively more distal positional identities. Nerves are not involved in establishing PD axial patterning, since aneurogenic limbs of reversed PD polarity regenerate distally in the same way as reversed innervated limbs (Wallace, 1980).

Another mechanism of PD pattern generation (Fig. 9C) postulates that RA specifies the stylopodium (assuming amputation at the proximal stylopodial level). A factor(s) contributed by the AEC provides an environment within which an autonomous timing mechanism specifies the positional identities of the zeugopodium and autopodium. There is evidence that RA specifies the stylopodium of the chick limb bud, whereas a timing mechanism, similar to the progress zone model developed by Summerbell, Lewis, and Wolpert (1973), specifies the zeugopodium and autopodium (Rosello‐Diez & Torres, 2011; Saiz‐Lopez et al., 2015). Such a mechanism would explain why the stylopodium is able to regenerate after grafting a mature hand to the mid‐stylopodium of axolot limbs, whereas more distal structures fail to be intercalated (Bryant & Iten, 1977; Pescitelli & Stocum, 1981), and why the stylopodium is not regenerated when undifferentiated blastemas derived from the distal stylopodium are grafted to the dorsal fin (Stocum, 1968a) or when supernumerary limbs are evoked from the stylopodium in the Lheureux model (Makanae, Mitogawa, & Satoh, 2014b). Also explained would be why BMP2 or 7 can stimulate adult mouse digits amputated through the second phalange to complete that phalange but not regenerate the distal‐most phalange, and stimulate neonatal mouse forelimbs amputated through the mid‐zeugopodium to regenerate the zeugopodium but not the autopodium (Ide, 2012; Masake & Ide, 2007; Yu, Han, Yan, Lee, & Muneoka, 2012; Yu et al., 2010). Clearly, there is much more to learn about blastema patterning from investigation of these phenomena.

These three models view the specification of PD positional identities after simple amputation as taking place serially, in proximal to distal order. This view fits the spatial and temporal pattern of expression during blastema growth of Hoxa9, 11 and 13 (Ohgo et al., 2010; Roensch et al., 2013; Tamura, Ohgo, & Yokoyama, 2010), which are thought to specify PD pattern in the limb bud (Izpisua Belmonte, Ede, Tickle, & Duboule, 1992; Yakushiji, Suzuki et al., 2009; Yokoyuchi, Sasaki, & Kuroiwa, 1991).

By contrast, Maden (1977) proposed a non‐serial PD specification model after simple amputation based on the intercalation of positional identities between proximal and distal boundary values (Fig. 9D). The proximal boundary is the fibroblast positional identity of the amputation level. The distal boundary might be conferred on initial fibroblast‐derived blastema cells by virtue of their contact with the AEC (Maden, 1977; Nye, Cameron, Chernoff, & Stocum, 2003). Confrontation of the two boundaries initiates an averaging cascade of intercalation. The first averaging event leads to intercalation of the positional identity halfway between the autopodium and the level of amputation. There are now three positional identities, and progressive mitosis and intercalary averaging continue to fill in the nearest neighbor map. There is evidence from mapping experiments that compartments representing the different limb segments are already present in the very early chick limb bud (Dudley et al., 2002; Stark & Searls, 1973) and in the urodele limb regeneration blastema (Echeverri & Tanaka, 2005). The problem is how to reconcile the proximal to distal sequence of expression of the Hoxa9−13 genes. The assumption would have to be made that serial expression of these genes does not adequately reflect the actual patterning mechanism itself, raising the speculation that expression of these transcription factors could be the result rather than the cause of the patterning mechanism.

#### 5.6.2 Distalization during intercalary regeneration

Autografting a distally derived blastema to a more proximal limb stump results in a delay in the growth and development of the grafted blastema, probably due to the time required for the tissues at the proximal limb level to undergo histolysis and contribute additional blastema cells, and to sufficiently re‐innervate the blastema. The grafted blastema then differentiates an autopodium according to its origin, and the cells contributed from the proximal host differentiate into the missing intermediate structures (Iten & Bryant, 1975; Maden, 1980b; Pescitelli & Stocum, 1980; Stocum, 1975). Intercalary deletions were the result when unirradiated distal blastemas were grafted to irradiated proximal stumps (Maden, 1980b). Proximal blastemas grafted distally developed according to origin, giving serially duplicated limbs (Iten & Bryant, 1975; Stocum & Melton, 1977), although Maden (1980b) found that 20% of cases showed intercalary regeneration from the graft that might have been of reversed polarity.

**Figure 10:**
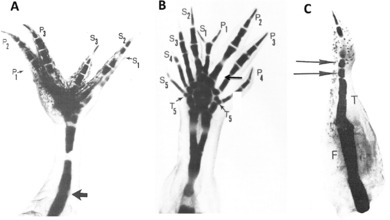
(A) Normal wrist blastema homografted from a dark axolotl to the double anterior stylopodium of a white animal. The graft regenerated with three forelimb digits (P1−P3) and evoked three supernumerary digits (S1−S3). A symmetrical double femur and tibia was intercalated from the host. Arrow, knee joint. (B) Normal wrist blastema of a white axolotl autografted to the ipsilateral double posterior stylopodium of the hindlimb. The graft formed four forelimb digits (P1−P4) and evoked five supernumerary digits (S1−S5). Primary and supernumerary sets of basipodial elements were regenerated. T5, tarsal 5. Arrow, symmetrical fibulae intercalated from host stump. (C) Double anterior wrist blastema homografted from a dark axolotl to the double anterior stylopodium of a white axolotl. The graft formed two carpals (arrows) and a single digit. A symmetrical distal femur (F) and tibia (T) were intercalated from the host stump. After Stocum (1980b, 1981)

Normal distal zeugopodial blastemas of axolotls grafted to a double anterior stylopodium evoke intercalary regeneration of a symmetrical distal stylopodium plus a single symmetrical element formed by the midline fusion of two anterior zeugopodial elements (Fig. 10A). Normal distal blastemas grafted to double posterior stylopodia more often evoke intercalation of a symmetrical distal stylopodium plus separated double posterior zeugopodial elements (Fig. 10B). In both cases, the graft develops as a normal autopodium while evoking supernumerary digits where anterior tissue confronts posterior tissue. A double anterior zeugopodial blastema grafted to a double anterior stylopodium (Fig. 10C) develops with the converged pattern of a single digit, and an intermediate symmetrical distal stylopodium and anterior zeugopodial element is intercalated between the two (Stocum, 1980b).

Collectively, these results suggest that each half of a double anterior stylopodium contributes to half of a symmetrical PD‐intercalated stylopodial and zeugopodial skeletal element, and that no interaction of anterior tissue with posterior tissue is required for blastema cell proliferation/distalization during intercalary regeneration. In contrast to what happens after simple amputation of a double anterior stylopodium, the intermediate blastema cells ignore their similarity in anterior positional identity and respond only to the discontinuity in PD identities between graft and host levels. Thus the mechanism of PD outgrowth and patterning seems to be different in double anterior stylopodia regenerating after simple amputation as opposed to intercalation and, by extension, between the regeneration of normal limbs after simple amputation versus intercalation when distal blastemas are grafted proximally. AP and proximal−distal positional identity seem to be uncoupled during intercalary regeneration. The difference might reflect the geometry within which each form of regeneration takes place, a hyperbolic cone for “normal” regeneration that directs circumferential cells centripetally to interact versus an elliptic cylinder requiring only intercalation of missing PD positional identities. Such geometrical influences on limb regeneration have not been explored. We cannot rule out, however, that there needs to be a DV differential for distalization to take place. This possibility might be tested by constructing stylopodia and zeugopodia that are double dorsal or double ventral, as well as double anterior, to see the effect on regeneration after simple amputation or after grafting double dorsal or double ventral blastemas to double dorsal and ventral limb stumps.

Meinhardt (1983b) explained the results of grafting a normal zeugopodial blastema to a normal or a double anterior stylopodium in terms of the boundary/bootstrap model, but this model cannot explain the intercalary regeneration that occurs after grafting a double anterior zeugopodial blastema to a double anterior stylopodium because there is no AP boundary in such constructs. The averaging model of Maden (1977), however, can account for intercalary regeneration in all these constructs because, in this model, the basal positional identity of the graft can act as a distal boundary confronting a proximal boundary represented by proximal host level blastema cells.

### 5.7 A multiple mechanism model of pattern formation

The foregoing models partially explain various aspects and observations on pattern formation in limb regeneration that reflect Lewis Held's (1992) apt description of patterning as the “Gordian knot of developmental biology.” We would like to have a unified model that accounts for all our observations on limb regeneration. It may be, however, that the urodele limb has multiple mechanisms of replacing structural loss and that one or another of these mechanisms predominates according to the type of tissue rearrangement experienced. Thus, elements of all the models described may be in play depending on the experimental circumstances and when combined would provide a framework for blastema patterning that explains most of the experimental observations.

This idea first supposes that the blastema is an anatomical mosaic of cellular contributions from each limb quadrant (Stocum & Cameron, 2011, for a review). As outlined above, experiments on half and double half limbs have revealed differential contributions of limb halves to the blastema. Maden (1982b), Maden and Mustafa (1982a), and Tank (1981) demonstrated that the mixed‐handedness supernumeraries evoked after APDV blastema rotation can be explained as mosaics that reflect the relative numbers of cells contributed by graft and stump tissues at the site of supernumerary formation and the polarities of these tissues with respect to one another. Maden and Mustafa (1984) analyzed the cellular contributions of graft and stump to the four classes of supernumeraries evoked by grafting APDV inverted triploid blastemas to diploid limb stumps. Each class of supernumerary was composed of a different percentage of graft and host cells, indicating the relative contribution from each.

Not all the results were explainable by mosaic cellular contribution, however. Amputated constructs of mixed‐handed axolotl zeugopodia (half normal, half inverted in the AP axis) regenerated not just the expected mixed‐handed limbs, but the same classes of anatomical patterns as after APDV inversion (Holder & Weekes, 1984; Muneoka et al., 1986a). Using triploid/diploid halves to make the constructs revealed a directionally biased intercalation as well as cell mixing (Muneoka et al., 1986b), which was also observed after limb bud tip inversions by Thoms and Fallon (1980). These results suggest a model in which both mosaic contributions to the blastema and intercalation to fill in gaps are at work. We must also consider the ability of blastema cells to sort out according to position as a factor in the final anatomy of regenerates. Wrist and elbow blastemas grafted to the stump/blastema junction of a hindlimb regenerating from the mid‐femur level do not develop according to origin at this location, or intercalate a set of supernumerary intermediate limb structures, but rather sort to their comparable position on the hindlimb regenerate where they develop according to origin (Crawford & Stocum, 1988a). These considerations suggest a flexibility in the choice of mechanisms used to re‐establish a normal neighbor map.

We might gain further insights by systematically mapping the contributions of the different quadrants of the limb cross‐section to the regenerates formed after APDV blastema rotation, double half limb regeneration, and intercalary regeneration, using GFP‐marked blastema or stump tissues, examining gene expression during intercalary regeneration, and systematically analyzing the molecular differences in positional identities.

## 6 BLASTEMA PATTERNING: AUTONOMOUS OR INDUCED?

The development of many embryonic tissues/organs and the homeostasis and regeneration of adult tissues is driven by self‐organizational mechanisms (Vogg, Wenger, & Galliot, 2016, for a review). This self‐organizational feature has enabled the creation of organoids (mini‐organs) from embryonic cells and iPSCs to investigate the development of cancer, to screen drugs for toxicity and therapeutic value, and to replace damaged or missing tissues (Clevers, 2016; Tsuji, 2017). The early limb regeneration blastema can be viewed as an in vivo organoid that gives rise to exactly those limb parts that were amputated. A major question argued over the course of the past century, however, is whether the blastema is self‐organizing, possessing all the information required to generate the pattern of the regenerate, or does it have a range of developmental plasticity, with the pattern imposed by signals from adjacent differentiated tissues? Put another way, is the early blastema a blank slate with regard to its developmental potency, or is its development restricted to its prospective significance? While the positional identity of blastema cells can be manipulated by RA, the question of how the pattern of the manipulated cells is organized remains the same.

Studies conducted from the 1920s to the 1940s reported that young limb blastemas grafted ectopically failed to develop, whereas older blastemas and young blastemas with stump tissue included in the graft were able to develop normally. Furthermore, early tail blastemas transplanted to limb stumps and vice versa were reported to develop according to host rather than donor origin, whereas older blastemas or early blastemas with stump tissue always developed according to donor origin. The polarity of regenerates formed by axially reversed early blastemas conformed to that of the host limb stump, whereas older blastemas formed regenerates that maintained their original polarity. These results suggested that the early blastema had no developmental capacity (was “nullipotent”) and that appendage type, position of origin, and axial polarity were determined by signals from adjacent stump tissue (Stocum, 1984, for a review).

Other investigators, however, pointed out that blastemas grafted ectopically with or without stump tissue might not survive, but stump tissuue would allow re‐formation of the blastema and that, in the absence of graft markers, the possibility could not be ruled out that conversion of one appendage type to another was due to a failure of the graft to survive and its replacement by host cells (Polezhaev, 1937). Early limb and tail blastemas grafted to lentectomized eyes were reported to develop as lenses (Schotte & Hummel, 1939), but these results were later shown to be an artifact of regeneration from host tissues (Stone, 1966). Early tail blastemas grafted to the developing embryonic ear region failed to form an otic vesicle (Emerson, 1940), but eye cups or otic vesicles grafted into blastemas were reported to induce lens or precartilage otic capsules from blastema cells. Again, however, the lack of markers in these experiments made it impossible to tell whether these structures were derived from graft or host cells. These kinds of experiments bear repeating using today's transgenic markers. Intriguingly, newt heart cardiomyocytes, which have the ability to dedifferentiate and regenerate injured heart tissue (Nag, Healy, & Cheng, 1979; Oberpriller & Oberpriller, 1974), dedifferentiated and transdifferentiated to skeletal muscle and chondrocytes when transplanted into the limb regeneration blastema (Laube, Heister, Scholz, Borchardt, & Braun, 2006), suggesting that the blastema environment can exert a powerful influence on cell phenotype that extends beyond limb cells.

**Figure 11:**
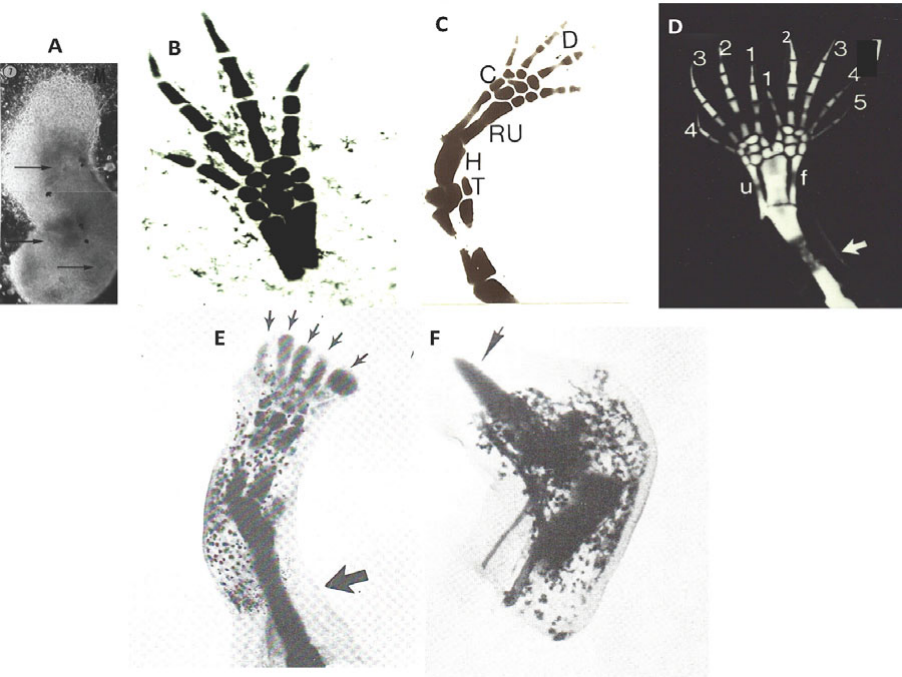
Autonomous development of the blastema. (A) Medium bud blastemal mesenchyme after 21 days of hanging drop culture. The blastema underwent abortive morphogenesis. Arrows point to dark shadows within the cell mass that reflect the development of a primitive cartilage. (B) Medium bud forelimb stylopodial blastema autografted to dorsal fin. (C) Proximal half of a palette stage fore limb stylopodial blastema autografted to the ankle level of the hind limb. The graft dedifferentiated and developed as a fore limb according to its level of origin. T = host tarsals; H = humerus; RU = radius/ulna; C = carpals; D = four digits. (D) Normal fore limb stylopodial blastema homografted to the same level of a double posterior hind limb stylopodium. Arrow = original graft‐host junction. The primary regenerate is forelimb (digits 1−4 on the left) with forelimb basipodial elements and radius/ulna (u). A supernumerary hindlimb regenerated with tibia and fibula (f) and digits 1−5. (E) Medium bud stylopodial blastema homografted from normal limb of a dartk axolotl to the same stylopodial level of the double posterior hind limb of a white axolotl. Large arrow = graft/host junction. The graft developed as a normal forelimb. The graft‐derived tissues suffered chronic immunorejection with dilation of blood vessels and hemostasis (small arrows) that sharply demarcated graft from host tissues. (F) Double anterior hind limb stylopodial blastema homografted distally to a double anterior hind limb zeugopodium. The blastema developed according to its double anterior stylopodial origin, forming a tapered cone of cartilage (arrow). After Stocum (1968a,b), Stocum and Melton (1977), Stocum (1980a), and Stocum (1981)

Other experiments have suggested that transplanted early blastemas can self‐organize according to origin if they receive adequate vascularization and innervation. Faber (1960) showed that stylopodial‐level medium bud axolotl blastemas marked with carbon particles in the prospective stylopodium and zeugopodium and grafted to the back failed to form proximal structures unless accompanied by stump tissues, but were capable of forming digits. He concluded that cells of the early blastema were intrinsically determined as autopodial structures, whereas the patterning of more proximal elements was determined by stump tissues. However, the carbon particles in this experiment were translocated into the back tissue, suggesting that prospective stylopodial and zeugopodial cells did not survive. Undifferentiated medium bud limb blastemas of *Ambystoma maculatum* larvae were capable of differentiation in vitro and of complete PD self‐organization from their level of origin after grafting them ectopically to a wound bed on the dorsal fin (Fig. 11A) (Stocum, 1968a, b).

Iten and Bryant (1975) reported that the handedness of regenerates formed after grafting distal early bud blastemas of adult newt forelimbs to the stylopodium with simultaneous reversal of the AP axis resulted in intercalary regeneration of intermediate PD structure, but with an AP digital pattern that either conformed to the host or more often had both host and graft (“intermediate”) handedness. Later stage blastemas developed with the handedness of origin. The formation of digits with intermediate handedness suggests that graft/host AP interactions resulting in supernumerary digit formation took place at the level of the autopodium as PD positional identities were filled in to that level. By contrast, blastemas grafted from a proximal to distal level most often conformed to their host level and developed only autopodial structures, suggesting an inductive activity of the stump. In both cases, histological examination of blastemas over the first few days after grafting suggested substantial survival of blastema cells. Stocum (1975) performed distal to proximal and proximal to distal grafts labeled with [^3^H]‐thymidine in *A. maculatum* larvae. Distal to proximal grafts resulted in normal limbs in which the autopodium was formed from the graft and intermediate structures were intercalated by cells from the host. Proximal to distal grafts appeared to conform to the host level and form only autopodium, but a later study in which the blastema was more accurately removed from its stump showed that the grafted blastema developed according to origin, forming serially duplicated structures (Stocum & Melton, 1977) (Fig. 11B). Intercalation does not take place after proximal to distal grafting, because the polarity of the gap is opposite to the polarity of the stump and graft. Grafted blastema mesenchyme forced to dedifferentiate when re‐covered by wound epidermis, as when several mesenchymes are massed and grafted to the back (De Both, 1970; Polezhaev, 1937), or when proximal halves of stylopodial forelimb blastemas are grafted to the ankle stump of the hindlimb (Stocum & Melton, 1977), developed according to origin. The mass of cells in these experiments is greater than that of an accumulation blastema and thus cell interactions may be a factor in whether or not the positional identity of individual blastema cells can be expressed (Stocum, 1984), although it should be noted that small clusters of prospective autopodial blastema cells failed to become incorporated into stylopodial tissue when transplanted into the prospective stylopodium of the blastema and instead sorted into the autopodial region (Echeverri & Tanaka, 2005). Pellets of posterior blastema cells cultured in vitro can induce supernumerary structures after implanting them to the anterior side of a blastema, but lose the capacity to do so after a week in culture (Groell, Gardiner, & Bryant, 1993).

Still other evidence for the early blastema as a self‐organizing system is that regenerate structure is not changed after grafting normal blastemas to double half limb stumps or vice versa (Fig 11C−E**)**. Furthermore, undifferentiated blastemas derived from forelimbs do not form hindlimb structures when grafted to hindlimb stumps and vice versa (Holder & Tank, 1979; Stocum, 1980a, b, 1981). Self‐organization is also consistent with the results of experiments on the early chick limb bud by Rosello‐Diez and Torres (2011). They showed that 100 μm thick stage 18 distal wing tips grafted to the level of the stage 21 prospective stylopodium were not induced to become stylopodium, but instead developed into autopodium whilst evoking intercalary regeneration of a zeugopodium from stylopodial mesenchyme.

Nevertheless, other experiments have led to different conclusions. Distal blastemas from axolotl limbs transgenic for GFP grafted to proximal limb stumps were reported to contribute to muscle and Schwann cell sheath proximal to their level of origin, leading to the conclusion that the early blastema is labile and is induced to form proximal structures by stump tissues in its new location (McCusker & Gardiner, 2013), as Faber (1960) had proposed. This conclusion is unwarranted, however, since Roensch et al. (2013) and Maden, Avila, Roy, and Seifert (2015) demonstrated that non‐fibroblastic cells do not carry positional identity and can abrogate the rule of distal transformation, whereas blastema cells derived from fibroblasts inherit level‐specific positional identity that ensures development of the blastema according to its level of origin and triggers intercalary regeneration of intermediate structures. McCusker and Gardiner (2013) also reported that the cells of early bud forelimb blastemas grafted to hindlimb stumps were induced to express higher levels of hindlimb‐specific *Tbx4* and early bud hindlimb blastemas grafted to forelimb stumps were induced to express higher levels of forelimb‐specific *Tbx5*, implying that blastemal plasticity extends to the conversion of forelimb blastemas to hindlimbs and vice versa. Hindlimb and forelimb blastemas do use similar positional information systems (Crawford & Stocum, 1988a, b), but converting one to the other should result in regenerates that each express the other's characteristic limb morphology and musculoskeletal structure. Such evidence has not been reported. Furthermore, the stability of the limb stump−blastema epigenetic code (Hayashi et al., 2015) argues against such a switch.

## 7 LINKING BLASTEMA GROWTH WITH PATTERNING

It is clear that blastema growth and distalization and patterning are coupled in some way. The mitotic index of the blastema during the maximum growth phase (medium bud through late bud) does not change until the start of redifferentiaton in either stylopodial or wrist blastemas, but the period of maximum growth is longer in stylopodial blastemas because there is more pattern to replace (Stocum, 1980c). Mitosis is inhibited in denervated medium bud blastemas, but these blastemas can nevertheless regulate to produce a miniature regenerate complete in the PD axis (Powell, 1969; Singer & Craven, 1948). This result indicates that very few mitotic divisions are required to set up the regenerate pattern, a conclusion backed up by the blastema mapping experiments of Echeverri and Tanaka (2005).

As to the molecular mechanism of patterning and morphogenesis, little is known. Bryant and Gardiner (2016) have advanced the hypothesis that the length of the G_0_/G_1_ portion of the cell cycle determines the size of the translatable transcripts that can be made by cells (”transcriptional gating”). The length of G_0_/G_1_ is different in different regions of developing or regenerating tissue, thus setting up a grid of different‐sized transcripts and proteins that represent the pattern. Some of these transcripts and proteins play key roles in transcriptional and proteomic networks that define positional identities, regulate cell division, and regulate cell differentiation. The role of growth factors, whether diffusable or tethered to the ECM, is to regulate in a concentration‐dependent way the length of the G_0_/G_1_ portion of the cell cycle. Bryant and Gardiner give several examples of spatial and temporal change in G_0_/G_1_ that is correlated with patterning, the most pertinent of which is the mouse limb bud, in which diferent regions of mesenchyme have variable lengths of G_0_/G_1_, and the inhibition of proliferation by RA during the time it takes to reprogram AP, DV, and PD positional identity, which possibly could involve a change in length of G_0_/G_1_.

These ideas have yet to be tested in regenerating limbs, but might be tested by performing single cell transcriptional profiling on small cell clusters from different parts of the blastema. They receive potential support from the regenerating rays of amputated zebrafish fins, which form blastemas at the tip of each ray. Each blastema is composed of several distal to proximal domains: a non‐proliferating distal blastema, and a proximal proliferating blastema surrounded by pre‐committed osteoblasts that differentiate as the blastema grows. The Wnt/β‐catenin signaling pathway is necessary for fin ray regeneration. Wnt signaling in the distal blastema of the ray has been found to set up organizing centers in the proximal blastema and epidermis that control epidermal patterning via Fgf and BMP signaling, and blastema proliferation by RA and hedgehog signals (Wehner et al., 2014). Whether there is a master organizer such as a Wnt/β‐catenin signaling region is unknown.

Another factor that deserves to be investigated with regard to growth and morphogenesis of the blastema is the potential role of convergent extension by mediolateral cell intercalation, a cell adhesion−traction mechanism that in the gastrula and neurula elongates the notochord and neural plate and tube (Keller et al., 2000). In addition to ECM deposition, convergent extension is likely to be operating during chondrocyte condensation in the limb regeneration blastema to elongate the skeletal elements of the emerging regenerate.

## 8 PROSPECTUS

The grand challenge of appendage regeneration is to achieve the regeneration of a human limb through what has been called “regenerative engineering” (Laurencin & Nair, 2016). Research on urodele limb regeneration will continue to inform this challenge, especially through comparative studies between urodele and regeneration‐deficient anuran limbs (Rao et al., 2014) and between position‐specific differences in the regenerative ability of mammalian appendages (Simkin, Sammarco, & Dawson, 2015). Why amputated urodele appendages regenerate and amputated anuran and mammalian appendages regenerate hypomorphically or not at all has been a long‐standing evolutionary question. Jazwinska and Sallin (2016) have proposed that the selective factor involved is the degree of functional demand placed on the appendages, this being higher in mammals and reflected in more complex structure and thus lower regenerative potential. The same structure/function/regeneration correlation extends to the urodele and zebrafish heart versus the mammalian heart. Furthermore, comparative studies suggest that differences in regenerative capacity and mechanism among species or different developmental stages are influenced by fundamental traits such as body size, aging, and growth pattern (Seifert et al., 2012), making it important to consider experimental results with reference to these traits, as has been shown for muscle contribution to the blastema (Sandoval‐Guzman et al., 2014).

Research on mammalian appendage regeneration will focus on understanding the roles of oxygen concentration, reactive oxygen species, manipulation of ECM degradation, BMP and Wnt signaling and the source of the cells that form the blastema in the amputated mouse digit. Several translational ideas have been proposed (Quijano, Lynch, Allan, Badylak, & Ahsan, 2015). First is that in‐depth understanding of the soluble factors involved in digit tip regeneration and their regulation can lead to the formulation of a molecular cocktail that initiates a regenerative cascade. The cocktail could be delivered by a bioreactor such as the Biodome, a thimble‐shaped device that can control pH, hydration, oxygenation, and electrical stimulation (Golding, Guay, Herrera‐Rincon, Levin, & Kaplan, 2016; Hechavarria, Dewilde, Braunhut, Levin, & Kaplan, 2010). One can even imagine making a tissue‐engineered limb construct that is parabiosed to the normal limb or other region of the body to become vascularized and develop to maturity.

An artificial blastema could be made by providing position‐specific human iPSC mesenchymal derivatives that either self‐organize into bone, cartilage, and muscle at the amputation plane or are guided in the formation of these tissues by vascularized scaffolds. Alternatively, dedifferentiation of position‐specific limb mesodermal cells could be induced in vitro to make a “natural blastema.” Aged human bone marrow mesenchymal stem cells subjected to specific three‐dimensional culture environments were reported to undergo dedifferentiation and autonomously form aggregates of cells resembling blastemas (Pennock et al., 2015). Dedifferentiation was associated with autonomously controlled autophagy that promoted cytoplasmic remodeling, mitochondrial regression, and a bioenergetic shift from oxidative phosphorylation to anaerobic metabolism. This bioenergetic shift has been previously observed in proteomic studies of regenerating urodele and anuran limbs (Rao et al., 2009, 2014). The role of metabolism in regeneration‐competent versus regeneration‐deficient limbs has been largely neglected since publication of the histochemical studies of Schmidt (1968) on newt limb regeneration. Bioinformatic analysis of functional molecular associations will also be a source of insight into the regulation of regeneration (Jhamb et al., 2011; King & Yin, 2016).

Another emerging idea is that, during development, organ and appendage fields set up a bioelectric code of ion channels/pumps and gap junctions that defines the pattern of the tissue and maps to the epigenetic code and pattern of gene activity associated with their development and regeneration. For tail and limb regeneration the bioelectric code is a memory system that reproduces the original code and thus the original transcription program and anatomical structure (Tseng & Levin, 2013). In a series of papers, Levin and colleagues have described the bioelectric code and provided extensive evidence for its existence and function. In addition, they have provided many examples of manipulation of the code that result in the rearrangement of large‐scale pattern (Levin, 2011, 2013; Mustard & Levin, 2014; Pezzulo & Levin, 2015). For example, a number of cell membrane channels associated with eye formation in *Xenopus* embryos induced eye formation in the gut, tail, or lateral plate mesoderm when misexpressed in these regions. Furthermore, the induction of H^+^ efflux/Na^+^ influx by a monensin‐containing cocktail initiated the whole cascade of events leading to tail regeneration during a regeneration‐refractory period of the tail bud during *Xenopus* development. Monensin is a polyether protein transfer inhibitor isolated from *Streptomyces cinnsmonensis*. The same cocktail was able to induce regeneration from stage 57 *Xenopus* tadpole limbs (Tseng & Levin, 2013). These regenerates formed digits with claws by 45 days after amputation through the mid‐tibia/fibula, although a more proximal structure was not regenerated, similar to the results reported by Yokoyama et al. (2001) using Fgf10 to stimulate stage 57 *Xenopus* limb regeneration.

The quest for regenerating a mammalian limb rests on the conviction that mammals retain latent ancestral genetic circuits for regeneration and we need only know how to activate them to regenerate a limb. This idea, however, has been challenged in an interesting way. Evolutionary surveys for Prod1, the receptor that integrates proliferation and patterning in regenerating urodele limbs, have found that Prod1 is unique to urodeles (Brockes & Gates, 2014; Garza‐Garcia, Driscoll, & Brockes, 2010; Geng, Gates, Kumar, & Brockes, 2015), suggesting that local selective forces have left only urodeles with the capacity for perfect limb regeneration. Therefore we must entertain the possibility that the genes involved in regeneration of anuran limb buds are insufficient for regeneration once the limb has differentiated, making it necessary to confer regenerative power on these and mammalian appendages by genetic engineering. For this, we might use the gene editing power of CRISPR/Cas9 to first regenerate anuran limbs as a proof of principle. Gene sequences key to urodele limb regeneration such as Prod1 plus others that might be lacking could first be introduced in vitro into the genome of anuran limb fibroblasts derived from the desired PD level of the limb. These edited fibroblasts could then be grafted in a fibrin clot (see Lin, Chen, & Slack, 2013) to the wound surface of recipient limbs and amputated at the same level from which the fibroblasts were derived. If these cells successfully support regeneration, the same could then be done with mouse digit/limb fibroblasts and, if successful there, with human limb fibroblasts.

An often‐asked question is how long it will take before we can regenerate a human limb. The answer is that we do not know, although we can envision that there will be a series of steps starting with regeneration of a digit. Some believe success is just around the corner. Others think we will never be able to regenerate such a large, complex structure as a limb. Such absolutes have been pronounced many times before and have been proven wrong. The beauty of basic science is that it can generate unexpected major advances over a short time frame; the production of iPSCs and the evolution of CRISPR/Cas combinations are two recent examples. The goal of human limb regeneration will rely on a convergence of ideas and research skills from many different scientific disciplines. In this, regeneration biologists will be racing bioengineers who are designing and building ever more sophisticated prosthetic limbs capable of neural interfaces (Carmena et al., 2013; Collinger et al., 2013; Lebedev & Nicolelis, 2006; Pedrocchi et al., 2013). Perhaps there will be a convergence of these two approaches to design a hybrid cyborg replacement appendage. Whatever happens, the regenerating urodele limb will continue to be an important source of insights into how we might regenerate human appendages.

## CONFLICT OF INTEREST

None
